# Glycan Profile and Sequence Variants of Certified Ricin Reference Material and Other Ricin Samples Yield Unique Molecular Signature Features

**DOI:** 10.3390/toxins16060243

**Published:** 2024-05-26

**Authors:** Roland Josuran, Andreas Wenger, Christian Müller, Bettina Kampa, Sylvia Worbs, Brigitte G. Dorner, Sabina Gerber

**Affiliations:** 1 Institute of Chemistry and Biotechnology, ZHAW Zurich University of Applied Sciences, 8820 Wädenswil, Switzerland; 2 Spiez Laboratory, Federal Office for Civil Protection, 3700 Spiez, Switzerland; 3 Biological Toxins (ZBS3), Centre for Biological Threats and Special Pathogens, Robert Koch Institute, 13353 Berlin, Germany

**Keywords:** ricin, *N*-glycosylation, sequence variants, cultivars, glycan profile

## Abstract

A certified reference material of ricin (CRM-LS-1) was produced by the EuroBioTox consortium to standardise the analysis of this biotoxin. This study established the *N*-glycan structures and proportions including their loci and occupancy of ricin CRM-LS-1. The glycan profile was compared with ricin from different preparations and other cultivars and isoforms. A total of 15 different oligomannosidic or paucimannosidic structures were identified in CRM-LS-1. Paucimannose was mainly found within the A-chain and oligomannose constituted the major glycan type of the B-chain. Furthermore, the novel primary structure variants E138 and D138 and four different C-termini of the A-chain as well as two B-chain variants V250 and F250 were elucidated. While the glycan proportions and loci were similar among all variants in CRM-LS-1 and ricin isoforms D and E of all cultivars analysed, a different stoichiometry for isoforms D and E and the amino acid variants were found. This detailed physicochemical characterization of ricin regarding the glycan profile and amino acid sequence variations yields unprecedented insight into the molecular features of this protein toxin. The variable attributes discovered within different cultivars present signature motifs and may allow discrimination of the biotoxin’s origin that are important in molecular forensic profiling. In conclusion, our data of in-depth CRM-LS-1 characterization combined with the analysis of other cultivars is representative for known ricin variants.

## 1. Introduction

Ricin is a potent plant protein toxin expressed in the seeds of the castor plant *Ricinus communis* (*R. c.*). The toxin is a member of the type II ribosome-inactivating protein (RIP) family and prevents 60S ribosomal subunit interaction with elongation factor 2, which leads to abolishment of protein biosynthesis [1]. The toxin is comprised of an A- and a B-chain that are linked by a disulphide bond. The N-terminal A-chain features an *N*-glycosidase activity specific for depurination of a single adenosine residue in the ricin/α-sarcin loop of the 28S ribosomal RNA [2,3], while the C-terminal B-chain facilitates binding and entry of the protein into the cell owing to its lectin property for galactosyl residues on cell surface receptors [4,5].

Four *N*-glycosylation motifs are found in the amino acid sequence of ricin; two sites are encoded in the A-chain at N10 and N236 and two of them are located in the B-chain at N95 and N135. Previous studies have reported *N*-glycosylation with paucimannosidic glycan at N10 of the A-chain and oligomannosidic-type *N*-glycosylation of the B-chain [6,7,8]. It is known that the second *N*-glycosylation motif of the A-chain at N236 is only partially occupied and either a paucimannosidic [9] or a oligomannosidic glycan structure [10] was described. Structural analysis of native-sourced full-length ricin revealed core glycan structures at both positions of the B-chain [11]. Two isoforms, ricin D and E, were isolated and characterised [12]. They share an identical A-chain [13], whereas the B-chain of ricin E is a chimera of B-chains of ricin D and *R. c.* agglutinin [14]. Various other ricin-like proteins are encoded by the ricin gene family that seem to be expressed at very low abundance in castor bean seeds [15,16].

In consideration of the demonstrated misuse of the toxin as bioterror and warfare agent in the past, its use is regulated in both the Chemical and Biological Weapons Conventions, prohibiting its production, dissemination, use and stockpiling [17,18,19]. Additionally, ricin is listed as a schedule 1 substance in the Chemical Weapons Convention [20]. To be prepared for potential incidents, a European consortium called EuroBioTox was initiated to establish, validate and train detection and identification methods for several relevant biotoxins within a pan-European network of expert laboratories and practitioners [21]. Certified ricin reference material (CRM-LS-1) was, inter alia, produced and characterised by laboratories of the project consortium. The glycosylation of ricin CRM-LS-1 is a specific characteristic of the product and its foreseen use as a certified reference material [22]. Here, we report the full glycan profile of ricin CRM-LS-1 and compare it to ricin samples from different origins. Our data confer unprecedented insights into the glycosylation patterns covering glycan structures, their relative shares, locus, and occupancy thereof, as well as the glycoforms of the toxin. Concomitantly, amino acid variations were elucidated and reported for the first time, yielding a novel degree of detail regarding the physicochemical attributes of native-sourced ricin and allowing improvement in the forensics of ricin.

## 2. Results

For characterisation of ricin CRM-LS-1, the full *N*-glycan profile as well as polypeptide variants were established. Carbohydrates were determined quantitatively with regard to monosaccharides, *N*-glycan structures and their loci within the protein and occupancy thereof, as well as with respect to the glycoforms. Furthermore, extensive analysis of heterogeneity within the primary structure was performed. A selection of the methods developed were additionally applied to a variety of ricin samples of different sources, isoforms and cultivars to compare their molecular properties.

**Figure 1:**
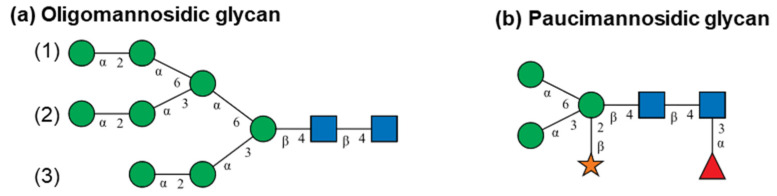
Schematic representation of (**a**) oligomannosidic and (**b**) paucimannosidic *N*-glycans following the Symbol Nomenclature for Glycans (SNFG) [24]. Green circles symbolise mannose, the orange star indicates xylose, the red triangle represents fucose and blue squares show *N*-acetylglucosamines. The lines illustrate linkage with positions indicated by numbers and configurations by Greek letters. The numbers in parentheses indicate the position of the antenna extended with outermost α1–2-linked mannose to distinguish multiple isomeric glycan structures. Nomenclature of glycan structures: (**a**) is M8 (1,2) and (**b**) is M3FX.

*N*-linked glycans show a conserved core structure in eukaryotes built from two *N*-acetylglucosamines extended by three mannoses at the non-reducing end. Glycans may be modified to establish oligomannosidic structures yielding three antennas (Figure 1a) or by a xylose or a fucose within the core structure producing paucimannosidic glycans (Figure 1b) [23]. The nomenclature applied by Kimura et al. [7,9] was used for designation of the glycans with the following convention for oligomannosidic- and paucimannosidic-type *N*-glycans found in plants: (i) the two core *N*-acetylglucosamines are omitted; (ii) oligomannosidic glycans are named Mn, where M stands for mannose and n is the number of mannoses linked to the two core *N*-acetylglucosamines; (iii) F indicates a core fucose that is α1–3-linked to the innermost core *N*-acetylglucosamine residue (the one that is attached to the asparagine side chain of the *N*-glycosylation sequence motif in the protein); (iv) X stands for the core xylose β1–2-linked to the first mannose after the two core *N*-acetylglucosamines; and (v) isomeric glycan structures with mannose attached to different antennas are labelled with the index of the antenna in parentheses. The two examples in Figure 1a,b are named M8 (1,2) and M3FX, respectively.

Peptides were labelled with the name of the protein (R stands for ricin, A for agglutinin), D or E for the different isoforms, the enzyme used for digestion (T stands for trypsin and C for chymotrypsin) and the chain (A or B) with indices of the first and the last amino acid counted from the N-terminus of the mature protein (e.g., RD-T:B(220–236)). The letter for the isoform was omitted in case the sequence was identical in both isoforms.

### 2.1. Glycosylation Analysis of Ricin CRM-LS-1

The strategy for glycosylation profile elucidation encompassed determination of monosaccharide composition in the first place followed by the identification of the structures and relative share of the glycans released from the protein. With the glycan structures being identified, combinations of theoretical masses of tryptic peptides containing a *N*-glycosylation motif and glycans were calculated and thus glycopeptides including the occupancies therein were assigned. Reversed-phase liquid chromatography-mass spectrometry (RPLC-MS) of intact A- and B-chains was applied to determine the combinations of glycans on each polypeptide chain yielding the glycoforms. Finally, the amount of sugar moieties was measured and the absolute quantity was determined using monosaccharide analysis.

#### 2.1.1. Monosaccharide Composition of Ricin CRM-LS-1 *N*-Glycans

**Figure 2:**
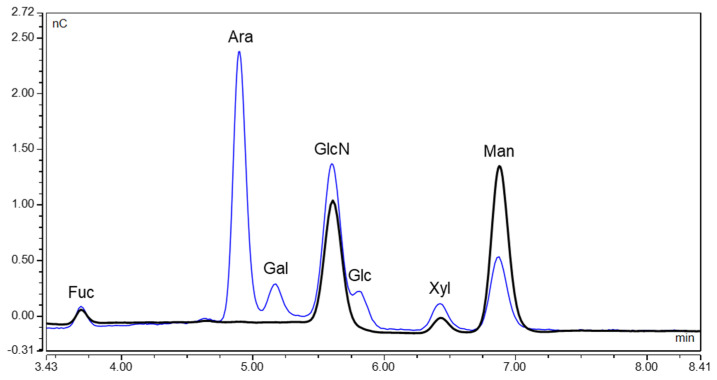
Monosaccharides released from ricin CRM-LS-1. HPAEC-PAD chromatogram of total hydrolysis (black) and overlayed chromatogram of a reference mixture containing fucose (Fuc), arabinose (Ara), galactose (Gal), glucosamine (GlcN), glucose (Glc), xylose (Xyl) and mannose (Man) at different concentrations (blue).

Monosaccharides were released from the protein by acidic hydrolysis and were analysed by high pH anion exchange chromatography with pulsed amperometric detection (HPAEC-PAD) for determination of monosaccharide composition and for absolute quantification (Figure 2). The four monosaccharides fucose (Fuc), *N*-acetylglucosamine, which was deacetylated to glucosamine (GlcN) during hydrolysis, xylose (Xyl) and mannose (Man) were detected in ricin CRM-LS-1. Neither arabinose (Ara) nor galactose (Gal) were identified; thus, ricin CRM-LS-1 does not feature any *O*-linked glycans since all known plant *O*-glycans contain at least one of the two monosaccharides [25].

#### 2.1.2. Identity and Relative Shares of Released Glycans from Ricin CRM-LS-1

**Figure 3:**
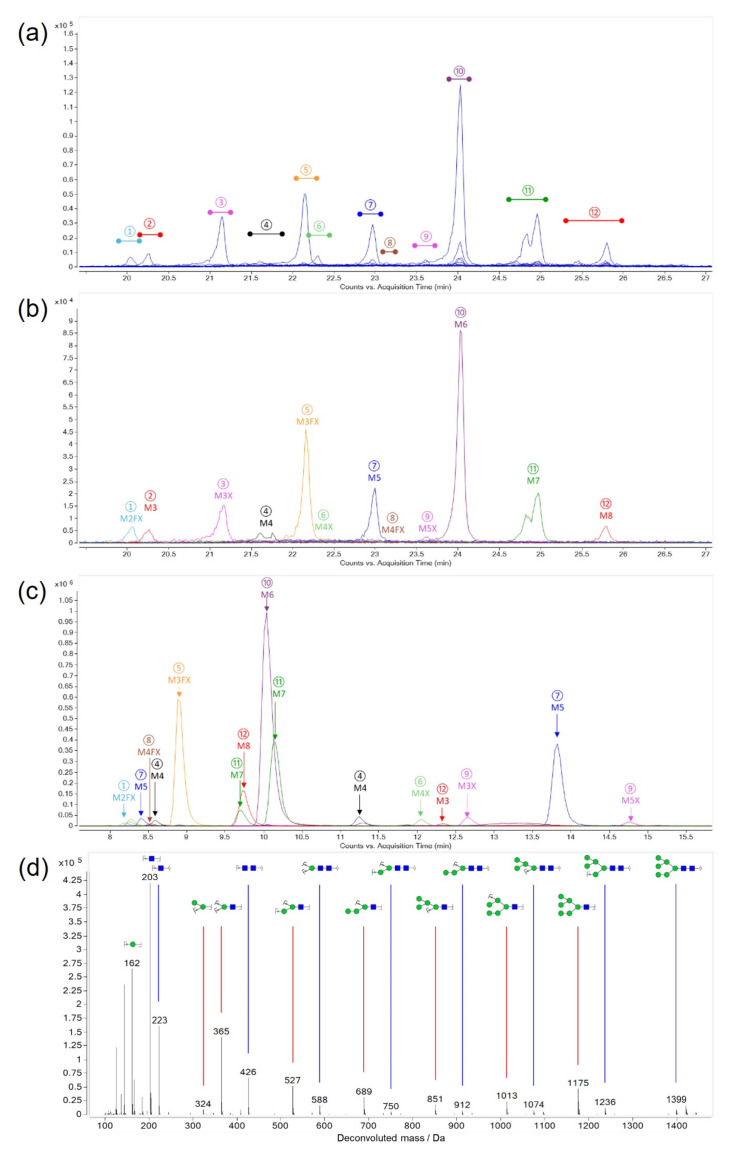
Identification of released glycans from ricin CRM-LS-1. Colours and labels indicate fractions taken from a consecutive run. (**a**) Extracted ion chromatograms of released glycans measured by HILIC-MS. (**b**) Overlay of extracted ion chromatograms of re-chromatographed fractions using HILIC-MS. Peaks labelled as M for mannose with the respective number of units and X for xylose. (**c**) Fractions from HILIC were analysed by PGCC-MS to separate isomeric glycans. (**d**) Representative, deconvoluted fragment spectrum of M6 with possible fragment structures assigned to mass peaks.

*N*-Glycans were released from CRM-LS-1 by hydrazinolysis to obtain the free reducing end. Further, the free amine groups of glucosamine were re-*N*-acetylated and the reducing ends were regenerated from β-acetohydrazide derivatives and finally reduced to the alditols. The glycans were first separated according to the monosaccharide composition using hydrophilic interaction liquid chromatography-mass spectrometry (HILIC-MS) and glycans were fractionated (Figure 3a). Twelve peaks containing glycans with different monosaccharide compositions were collected and re-chromatographed with the HILIC-MS method to evaluate the purity of the fractions (Figure 3b). Subsequently, the fractions collected were analysed using porous graphitised carbon chromatography-mass spectrometry (PGCC-MS) to separate isomeric glycans that coeluted in HILIC (Figure 3c). The separated glycans were fragmented by collision-induced dissociation to elucidate the monosaccharide sequence. As expected, two mannose ladders of specific lengths were detected, which were shifted by the mass of the reducing end GlcNAc. A representative deconvoluted spectrum for M6 is shown in Figure 3d. The linkage configurations were investigated using a combination of chromatographic, mass spectrometric and enzymatic approaches suggested by Pabst et al. [26]. M3, M5 (1,2) and M6 were identified by comparison with commercially available reference substances. The linkage constitution of the other glycan structures was established via specific enzyme reactions and comparing mass and retention times of the products with reference substances produced in-house as described in detail in the literature [26,27,28,29,30,31,32,33].

**Figure 4:**
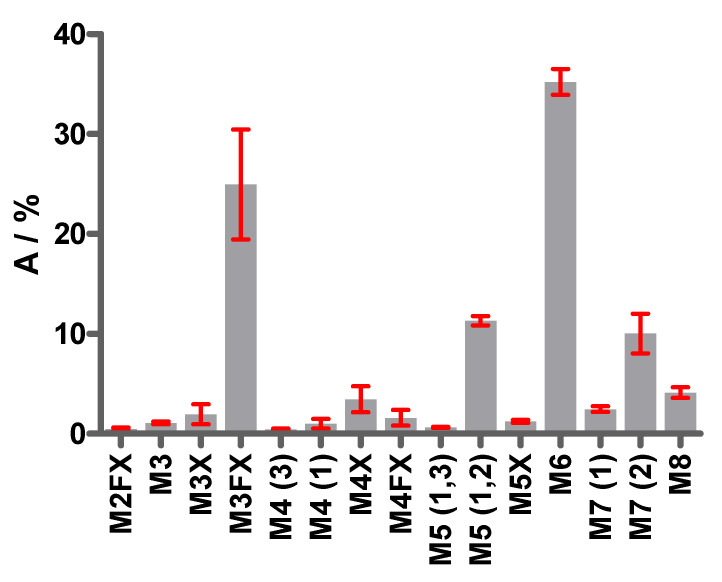
Proportions of *N*-glycans released from ricin CRM-LS-1. Bars represent the relative peak areas in extracted ion chromatograms with standard deviation of replicate analysis performed by two operators on different days in triplicate from different vials. M, mannose; F, fucose; X, xylose. The numbers of mannoses per glycan are indicated. The two conserved core *N*-acetylglucosamine (GlcNAc) moieties are not depicted in the glycan nomenclature used. The numbers in parentheses depict the antenna with terminal mannose that differs in constitution isomers.

Three fractions (4, 7 and 11) collected from HILIC (Figure 3a) showed two peaks in PGCC resolving two isomeric glycans for M4, M5 and M7 each (Figure 3c). The other nine fractions from HILIC revealed single peaks in PGCC. Thus, 15 different oligo- and paucimannosidic glycan structures were elucidated in ricin CRM-LS-1, representing common structures reported for plant protein *N*-glycosylation [34]. Furthermore, the proportions of the different glycans were elaborated by PGCC-MS without prior separation by HILIC. Sample preparation and measurement was performed by two different operators on different days and from three different original CRM-LS-1 vials each. The released and identified glycans as well as proportions thereof are represented in Table 1 and Figure 4.

The most abundant oligomannosidic glycans were M5 (1,2), M6 and M7 (2), which represented more than 50% of all glycan species. M3FX constituted the highest proportion of the paucimannosidic glycan species, accounting for 25% of the total. All other glycans had proportions below 10%.

#### 2.1.3. Locus and Occupancy of Glycans in Ricin CRM-LS-1

Ricin has four glycosylation motifs that are reported to be glycosylated, two in the A-chain at asparagine residues at positions 10 (A:N10) and 236 (A:N236) and two in the B-chain at the asparagine residues at positions 95 (B:N95) and 135 (B:N135) [7,9]. The released glycans from ricin CRM-LS-1 were identified and the proportions were measured by HILIC-MS and PGCC-MS. In the following, the glycans were mapped to the four glycosylation motifs and the occupancies of the loci were quantified. For this purpose, ricin CRM-LS-1 was digested with trypsin and chymotrypsin to obtain glycopeptides containing the four glycosylation motifs. The glycopeptides were analysed by HILIC-IM-MS. For identification of glycopeptide peaks, an extracted ion chromatogram (EIC) of the diagnostic fragment ion of GlcNAc (*m*/*z* 204) at elevated collision energy was generated [35]. The mass of the precursor ions in these peaks were matched with the calculated mass of the peptides containing the glycosylation motif plus the detected glycans. Tryptic glycopeptides were employed for the evaluation of glycosylation sites A:N10, A:N236 and B:N95 and the chymotryptic glycopeptide was used for B:N136. Also, the corresponding non-glycosylated peptides were detected with HILIC-IM-MS accordingly, except for the tryptic peptide with the glycosylation site A:N236 with the sequence NGSK due to an isobaric and co-eluting peptide SNGK from the B-chain. Both peptides were identified by tandem MS analyses but could not be quantified. Since the occupancy of A:N10 was found to be virtually 100% as discussed below, the proportion of non-glycosylated A:N236 was determined by mass spectrometric analysis of the intact A-chain. The ratio of peak areas in the deconvoluted mass spectrum of the doubly and the singly glycosylated A-chain was employed to calculate share of glycoforms, as explained in Section 2.1.5.

**Figure 5:**
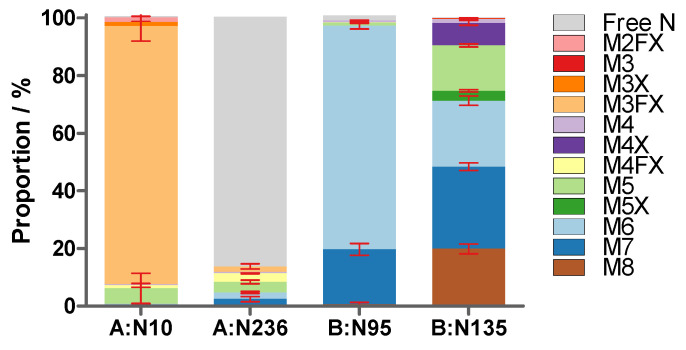
Glycan proportions and site occupancy (peak area/%) in ricin CRM-LS-1 at the four glycosylation motifs within the two polypeptide chains (A:N10, A:N236, B:95 and B:N135) determined with the glycopeptides. Values with standard deviation (red error bars) were acquired using HILIC-IM-MS of tryptic and chymotryptic peptides from four independent sample preparations.

The occupancy of the four glycosylation sites was established from four independent sample preparations and is summarised in Table 2 and Figure 5. The A-chain was decorated to 99.5%, mainly with the M3FX paucimannosidic glycan at the glycosylation site A:N10, and revealed very low occupancy of a mixture of paucimannosidic and oligomannosidic glycans of only 13% at A:N236, whereas the major proportion at this latter position was non-glycosylated. However, both motifs of the B-chain were almost exclusively glycosylated with oligomannosidic glycans with three to eight mannose residues that showed partial xylosylation. Our finding of higher occupancy at motif NFT at position A:N10 compared to NGS at A:N236 and almost 100% site occupancy in the NXT motifs within the B-chain is in agreement with the literature reporting a generally lower occupancy within NXS motifs compared to NXT motifs [36].

Shares of released glycans (PGCC-MS) were compared to the relative share of glycopeptides with a specific glycan (HILIC-IM-MS) to demonstrate the robustness of the data obtained through orthogonal approaches (Supplementary Material Figure S1) and to further validate data from other ricin cultivars (below). Both methods yielded highly similar results without statistically significant differences.

#### 2.1.4. Quantification of Released Monosaccharides

**Figure 6:**
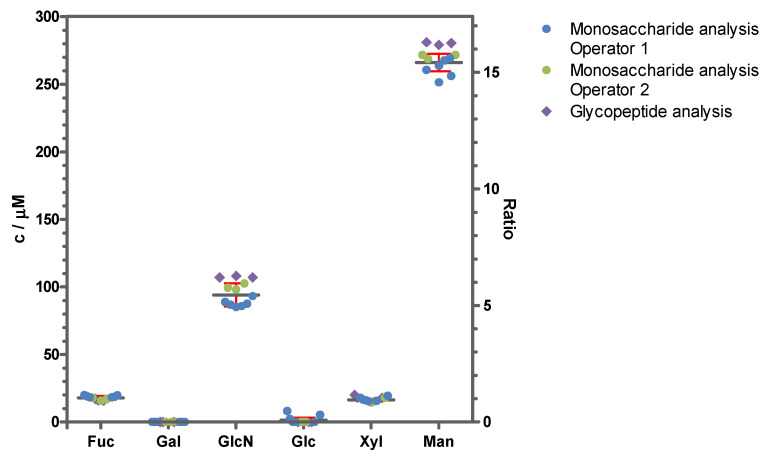
Monosaccharide content of ricin CRM-LS-1 as an absolute concentration and as monosaccharide-to-protein ratio. Monosaccharide concentrations (left axis) and molar monosaccharide-to-protein ratios (right axis) were measured with HPAEC-PAD of hydrolysed samples by two operators in a sixfold setup (operator 1, blue circles) and in a triplicate setup (operator 2, green circles) from a different vial each on a different day. Mean values and standard deviations were represented as a grey line and red whiskers, respectively. The monosaccharide-to-protein ratio was additionally calculated from the proportions of glycans at the four glycosylation sites acquired by glycopeptide analysis with HILIC-MS from three independent sample preparations (purple diamonds).

To validate the relative quantity of the glycans, monosaccharides were released by acidic hydrolysis and quantified by HPAEC-PAD (Figure 1). The concentration of released monosaccharides was measured by two operators in a sixfold setup (operator 1) and in a triplicate setup (operator 2) from a different vial each on a different day and the molar ratio of monosaccharides to protein was determined (Figure 6, Supplementary Material Table S5). The highest concentration was detected for mannose, a finding which met the expectation considering the high proportion of oligomannosidic glycans determined from the glycans released by PGCC-MS. *N*-acetylglucosamine was completely deacetylated to glucosamine during hydrolysis; therefore, the glucosamine concentration was assessed in order to quantify *N*-acetylglucosamine. In agreement with its conservation as a disaccharide within the core in all glycan structures, it showed the second highest concentration. The low shares of xylose and fucose also correlated well with the glycan data, since the highly abundant oligomannosidic glycans did not contain any of the two monosaccharides and their presence in paucimannosidic glycans either contained one xylose or one xylose and one fucose. A very low quantity of glucose was detected, a monosaccharide that was not contained in the glycans identified. Glucose constitutes the main component of cellulose and hence may likely be introduced into the sample from paper wipes [37]. The protein and monosaccharide concentrations of ricin CRM-LS-1 yielded the molar ratio of monosaccharide to protein. The data were consistent with the calculated composition of the glycopeptides as depicted in Figure 6 and Supplementary Material Table S5.

#### 2.1.5. Glycoforms of Ricin CRM-LS-1

Initially, the occupancy and the glycan structures at a specific glycosylation site of ricin CRM-LS-1 were elucidated. In a further step, mass spectra of the intact A- and B-chains of ricin were employed to determine the glycoforms identifying the combinations of glycan structures in each chain. Hence, completely reduced and alkylated ricin CRM-LS-1 was chromatographed, and the mass spectra were acquired using RPLC-MS. A single peak at 14.89 min containing the B-chain of ricin D and a double peak at 17.25 min and 17.37 min with differently processed ricin D A-chains were detected. The identities of the A- and B-chains were confirmed by the mass of the deconvoluted peak spectra and by analysing the tryptic digest after fractionating the peaks using RPLC-MS (Supplementary Materials Tables S1 and S2).

**Figure 7:**
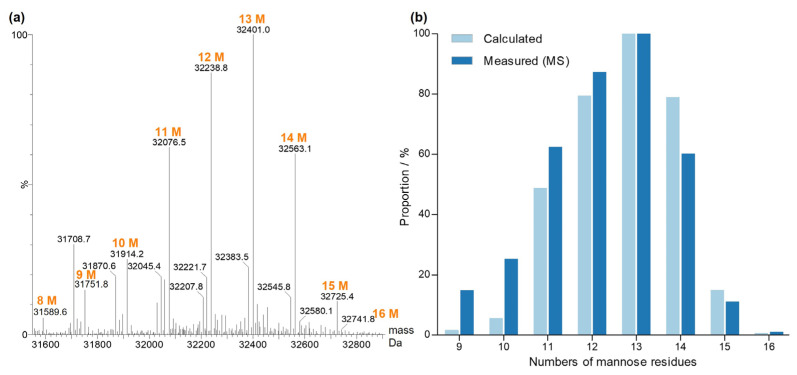
Glycoforms of the ricin CRM-LS-1 D B-chain. (**a**) Deconvoluted and centred mass spectrum. Orange labels depict the sum of the mannose residues of two glycans at the two glycosylation sites. The masses at 31,708.7 Da and 31,870.6 Da are cleavage products of the B-chain. (**b**) Number of mannose residues of *N*-glycans attached to the B-chain. Measured intensities (dark blue) were compared to the calculated proportions (light blue) of glycan combinations.

The abundance of all different combinations of the determined *N*-glycans at the two glycosylation sites of the B-chain eluting at 14.89 min were calculated and matched with the experimental deconvoluted mass spectrum (Figure 7). The mass spectrum showed four main mass peaks corresponding to the mass of the B-chain with a combination of two glycans with sums of 11 to 14 mannose residues (32,076.5 Da, 32,238.8 Da, 32,401.0 Da and 32,563.1 Da). This sum was consistent with the combinations of the most abundant glycans M6 and M7 for the glycosylation site B:N95 and M5, M6, M7 and M8 for the glycosylation site B:N135. Signals of lower intensity were found for combinations with less than 11 and more than 14 mannose residues. However, species with a lower mannose number were slightly overestimated in the mass spectrum, probably due to loss of terminal mannoses caused by in-source decay. The theoretical distribution of the sum of mannose residues at the two *N*-glycosylation sites of the B-chain was calculated based on the proportions of glycans determined in Section 2.1.3, assuming that both sites were glycosylated independently from each other (Figure 7b). The good match of the calculated and the measured peak distribution confirmed the validity of the established proportions of the *N*-glycans.

Signals of lower intensity of xylosylated oligomannosidic glycans of the forms M4X and M5X were previously detected at the glycosylation site B:N135 by glycopeptide analysis as described in Section 2.1.3. The most abundant glycoform of the B-chain containing xylose corresponded to M6 at B:N95 in combination with M4X at B:N135, resulting in 10 mannoses plus one xylose matching with the mass peak at 32,045.4 Da. Consequently, mass peak at 32,207.8 Da corresponded to a xylosylated glycoform.

**Figure 8:**
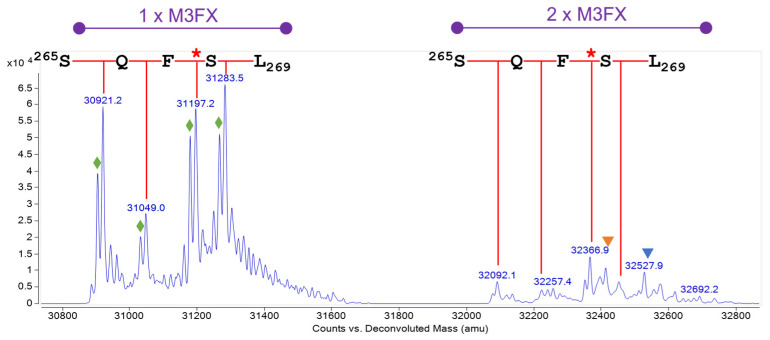
Glycoforms of the ricin CRM-LS-1 D A-chain. Deconvoluted mass spectrum. Two peak clusters with singly and doubly glycosylated A-chain are depicted in purple. The red callipers illustrate the different C-termini of the A-chain, amino acid residues are stated in single letter code, and the red stars mark the published C-terminus comprising F267. Orange and blue triangles mark the mass peaks of the A-chain with M3FX plus M5 or M4FX. Green diamonds designate the E138D sequence variant as elaborated in Section 2.2.3.

For the A-chain, the combined and deconvoluted mass spectrum over the whole A-chain species showed one peak cluster in the mass range from 30,800 Da to 31,400 Da with a singly glycosylated form and another peak cluster within the mass range from 32,000 Da to 32,800 Da with a doubly glycosylated form (Figure 8). The double peak in the chromatogram did not arise from these two glycoforms but was produced due to different amino acid variants as described below. Since the glycosylation site A:N10 only showed 0.5% of free asparagine, the lower mass peak cluster corresponded almost exclusively to the singly glycosylated form with M3FX with occupancy of 89.5% at site A:N10, whereas the peak cluster at higher mass revealed ricin A-chain with glycosylation at both glycosylation sites; a finding which is consistent with the literature [9,38]. The most abundant glycans at glycosylation site A:N236 were M3FX, M4FX and M5, the masses of which were in agreement with data obtained for the glycopeptides.

The mass peak at 31,197.2 Da corresponds to the A-chain glycosylated with one M3FX and the published amino acid sequence [39,40,41]. Three further mass peaks (30,921.2 Da, 31,049.0 Da and 31,283.5 Da) were detected, revealing four variable C-termini of the A-chain, devoid of either F267 or both F267 and Q266, and one with an additional S268 compared to the published C-terminus. The three A-chain variants with a polar amino acid at the C-terminus eluted in a single peak at 17.25 min, whereas the variant with the hydrophobic phenylalanine eluted shortly afterward at 17.37 min, generating a double peak. These four different C-termini were also confirmed by RPLC-IM-MS and HILIC-IM-MS analysis of tryptic peptides (Supplementary Materials Tables S3 and S4).

### 2.2. Comparison of Ricin and Isolated Isoforms Thereof from Different Cultivars

CRM-LS-1 was produced and certified as a reference standard; hence, the comparison of it with ricin from other sources and other cultivars was of further interest. To this end, ricin CRM-LS-1 that was purified from *R. c*. ‘Zanzibariensis’ (CRM Zanz. 1) was analysed side by side with two other ricin preparations purified using a modified protocol (Zanz. 2) and in another laboratory (Zanz. 3), each from a different batch of seeds from *R. c*. ‘Zanzibariensis’. Furthermore, ricin was purified from *R. c*. ‘Carmencita’ (Carmencita), *R. c*. ‘Tanzania’ (Tanzania), *R. c*. ‘Gibsonii’ (Gibsonii), *R. c*. ‘Sanguineus’ (Sanguineus), *R. c*. ‘India’ (India) and *R. c*. ‘Impala’ (Impala) side by side to Zanz. 3. Two additional samples were produced with isolated ricin D (Carmencita D) and isolated ricin E (Carmencita E) from the identical batch of seeds from *R. c*. ‘Carmencita’ to evaluate the differences between ricin D and ricin E.

#### 2.2.1. Occupancies and Relative Shares of *N*-Glycans

**Figure 9:**
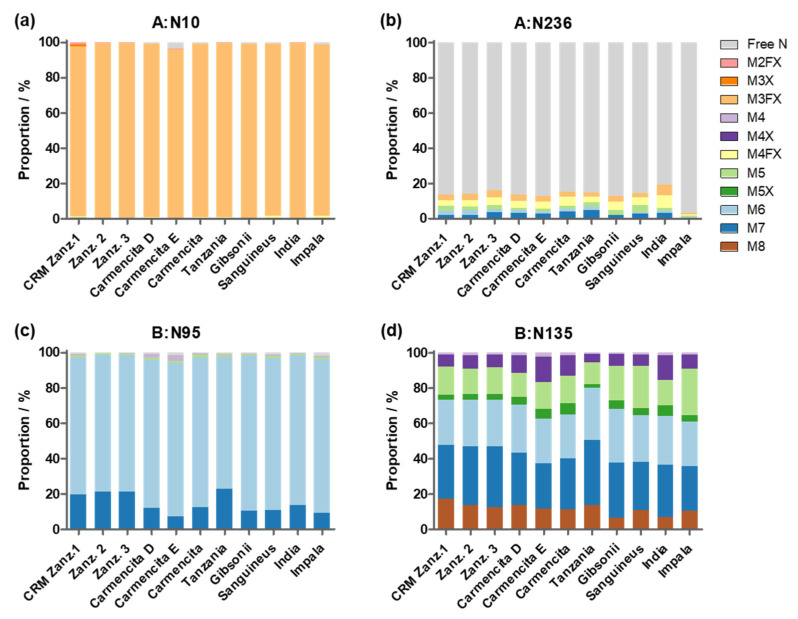
Proportion of glycan structures attached to the four *N*-glycosylation motifs of ricin, comparing ricin CRM-LS-1 (Zanz. 1) to ricin purified from other cultivars and to different ricin isoforms determined in single measurements. Glycosylation sites for (**a**) A:N10, (**b**) A:N236, (**c**) B:N95 and (**d**) B:N135 as shown above each histogram. Ricin was purified from three different batches of *R. c.* ‘Zanzibariensis’. Ricin D and E were isolated from *R. c.* ‘Carmencita’. The other ricin samples were purified from different cultivars as stated below the graphs.

The orthogonal glycan analysis for ricin CRM-LS-1 confirmed our accurate glycan profile data. Samples of all the above-mentioned ricin materials were digested with trypsin, chymotrypsin or Lys-C and analysed with HILIC-IM-MS to measure the glycosylation at the four glycosylation sites. As shown for ricin CRM-LS-1, the proportion of non-glycosylated asparagine at A:N236 could not be measured using the tryptic peptide due to the presence of an isomeric peptide deriving from the B-chain. Instead, ricin was digested with Lys-C to reduce the complexity caused by the four different C-termini and the occupancy of A:N236 was measured using RPLC-MS. The glycan structures at the four glycosylation sites of ricin from the different sources analysed are represented in Figure 9 and in Supplementary Materials Tables S6–S9.

We found that all analysed samples showed very similar glycosylation profiles, independent from the batch of seeds (Zanz. 1–3), the isoform (Camencita D or E) or the cultivar. Site N10 of the A-chain showed highly abundant glycosylation with M3FX of more than 96% in the different samples. The second glycosylation motif at A:N236 was occupied with either M3FX, M4FX, M5 or M6 in all samples to very similar shares of 13% to 20%, except *R. c*. ‘Impala’, which had a very low occupancy of only 3%. No paucimannosidic glycans were detected in the B-chains of the ricin samples. B:N95 was occupied with oligomannose structures devoid of xylose, with M6 as the main form in all samples with a share of 75% to 87%. M7 was the second most abundant glycan in *R. c.* ‘Zanzibariensis’ and *R. c.* ‘Tanzania’ at 19% to 23%, whereas its proportion in all other cultivars accounted for only 7% to 13%. The second glycosylation site B:N135 was occupied with oligomannosidic glycans from M4 to M8 and two xylosylated oligomannose glycans M4X and M5X in all samples with different shares. Further replicate measurements would be required to determine the significance of these differences.

#### 2.2.2. Amino Acid Sequence Variations of A- and B-Chains from Different Cultivars and Isoforms

**Figure 10:**
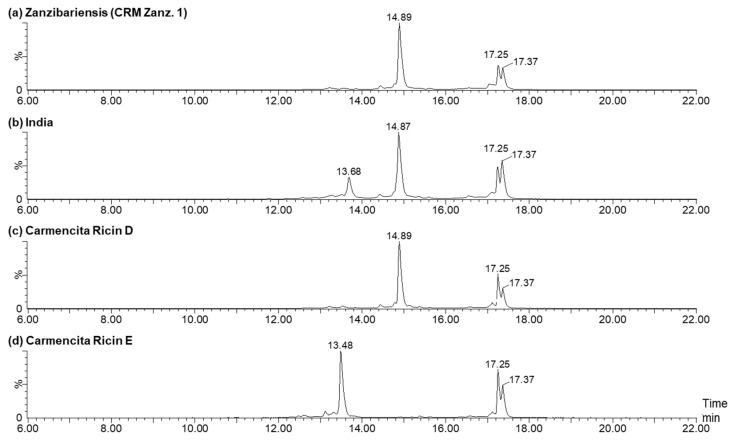
Ion chromatograms of A- and B-chain ions of ricin CRM Zanz. 1 (**a**) compared to ricin from other cultivars and isolated isoforms (shown in subfigures (**b**–**d**)) after reduction and alkylation applying RPLC-MS. The ricin A-chain eluted as a double peak at 17.25 min and 17.37 min, the B-chain of ricin D showed a mean retention time of 14.88 min and the B-chain of ricin E eluted at 13.68 min or 13.48 min. Ricin D and E were isolated from *R. c.* ‘Carmencita’ and are shown in (**c**,**d**).

The A- and B-chains of the different ricin cultivars and isoforms listed above were analysed by RPLC-MS and compared to ricin CRM-LS-1 (CRM Zanz. 1) (Figure 10). Ricin was reduced and alkylated to permanently dissociate the two chains for following separation and detection by RPLC-MS. Representative chromatograms showing specific A-chain and B-chain ions are depicted in Figure 10. The analysis yielded a highly reproducible elution of the A-chain as a double peak at 17.25 min and 17.37 min in all ricin samples investigated. While ricin from all cultivars excluding ricin CRM Zanz. 1 (Figure 10a) revealed B-chain mixtures of both isoform D and E eluting at 14.89 min, very similarly to the B-chain of ricin D isolated from *R. c.* ‘*Carmencita*’ (Figure 10c), and at 13.48 min, highly similar to the B-chain of ricin E isolated from *R. c.* ‘*Carmencita*’ (Figure 10d), the B-chain of *R. c.* ‘India’ revealed a B-chain signal at 13.68 min (Figure 10b).

**Figure 11:**
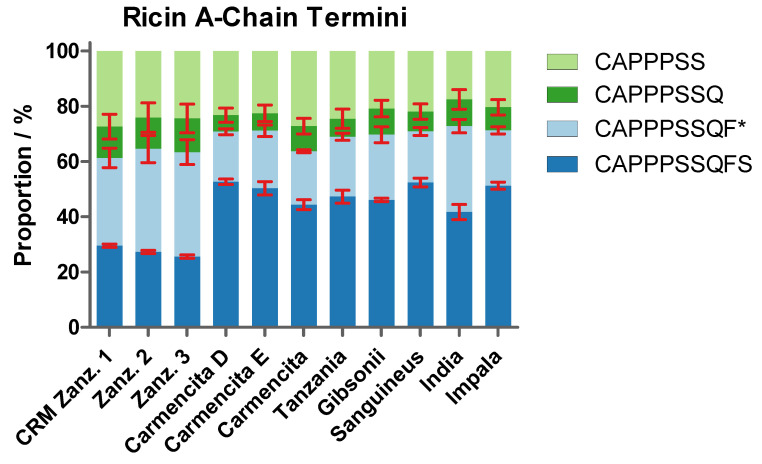
Proportions of different C-termini of the ricin A-chain of various cultivars and isoforms compared to CRM-LS-1 (CRM Zanz. 1). The origins of samples are stated below the histogram. Data obtained from average of three measurements, a single data set of intact A-chain acquired by RPLC-MS and C-terminal tryptic peptides analysed by HILIC-IM-MS measured in duplicate. Error bars indicate the standard deviation of the combined measurements. The asterisk indicates the peptide of the published C-terminus [13,40].

The four different C-termini of the ricin A-chain detected in ricin CRM-LS-1 producing the characteristic double peak were discovered in all ricin samples and the proportions thereof were determined in duplicate from a single sample preparation and calculated by the signals in deconvoluted mass spectra (Figure 11, Supplementary Material Table S10). Furthermore, the identities and proportions were confirmed by specific tryptic peptides applying HILIC-IM-MS (Supplementary Material Table S11). The data obtained from the intact A-chain produced very similar shares compared to the peptide analysis that validated our data by orthogonal methods. The average of these results is represented in Figure 11. CRM Zanz. 1 and the two additional ricin samples from *R. c.* ‘Zanzibariensis’ (Zanz. 2 and 3) yielded very similar shares for the four C-termini, demonstrating a highly conserved polypeptide chain processing within three ‘Zanzibariensis’ cultivars.

The longest variant of the A-chain (CAPPPSSQFS) was significantly less abundant in ricin purified from *R. c.* ‘Zanzibariensis’ compared to all other samples evaluated using the Bonferroni test. The share of the published C-terminus (CAPPPSSQF*) was significantly higher in ricin from *R. c*. ‘Zanzibariensis’ and *R. c*. ‘India’ than in ricin from the other cultivars. The A-chains from the cultivars of *R. c.* ‘Carmencita’, ‘Tanzania’, ‘Gibsonii’, ‘Sanguineus’ and ‘Impala’ featured very similar carboxy termini. No significant differences between the shorter two variants (CAPPPSSQ and CAPPPSS) were determined in all measured samples.

#### 2.2.3. Ricin A-Chain Amino Acid Sequence Variations

**Figure 12:**
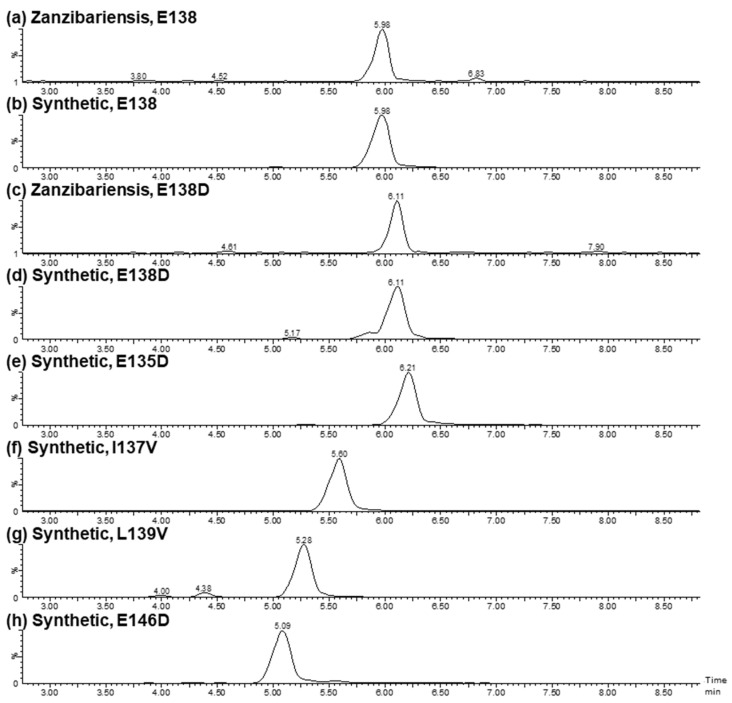
Verification of the amino acid variation E138D in the A-chain of ricin by retention time. Extracted ion chromatograms of the A-chain peptides from position 135 to 166 (R-T:A(135–166)) of tryptic-digested ricin purified from *R. c.* ‘Zanzibariensis’ (**a**,**c**) and chemically synthesised (**b**,**d**–**h**) with different amino acid substitutions. The amino acid sequence of (**a**,**b**) with E138 correspond to the sequence from the literature (^135^ENIELGNGPLEEAISALYYYSTGGTQLPTLAR_166_), whereas the amino acid substitutions thereof (**c**–**h**) are stated in the headers.

**Figure 13:**
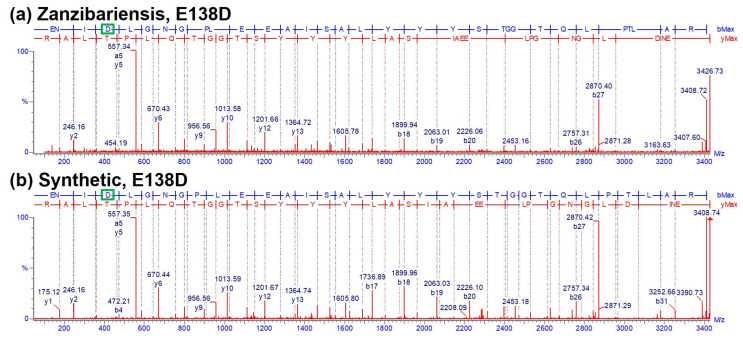
Verification of the amino acid variation E138D in the A-chain of ricin by tandem MS. Fragment ion spectra (CID) from tryptic A-chain peptide R-T:A(135–166) E138D compared to the synthetic peptide with an amino acid substitution of glutamate for aspartate at position 138 (highlighted by green boxes). (**a**) Spectrum of a tryptic peptide of ricin purified from *R. c.* ‘Zanzibariensis’; (**b**) spectrum of a synthetic peptide with the sequence ^135^ENIDLGNGPLEEAISALYYYSTGGTQLPTLAR_166_.

Two abundantly expressed ricin isoforms have been described to date, ricin D and ricin E, which are reported to be built of identical A-chains but differ partially in their B-chain amino acid sequences [14,42,43,44,45]. Mass spectrometric analysis of the reduced and alkylated A-chain of ricin purified from *R. c.* ‘Zanzibariensis’ and *R. c* ‘Tanzania’ showed additional mass peaks 14 Da lower than the calculated average mass of the published sequence of approximately 65% of signal intensity compared to the main peaks (Figure 8, green diamonds). Since, to the best of our knowledge, no post-translational modification of proteins resulting in a loss of 14 Da exists, a variation within a single amino acid position was expected to result in this additional mass peak. MS analysis of enzymatically and chemically hydrolysed ricin led to the conclusion that the amino acid variation was located in the tryptic peptide 136–166. The data further indicated a high probability of glutamate substitution for aspartate at position 138 (E138D). The tryptic peptide containing E138 and D138 (R-T:A(135–166)) and a set of five isomeric peptides with exchanged amino acids resulting in a loss of 14 Da were synthesised to clearly identify the locus and type of amino acid variation. RPLC-MS analysis of tryptic peptides from ricin purified from *R. c.* ‘Zanzibariensis’ showed one peak with the mass of the peptides with E138 at 5.98 min (Figure 12a) and one peak with the mass of the peptide with E138D at 6.11 min (Figure 12c). The synthetic peptides of these two sequence variants, E138 and D138, showed precisely the same retention times and masses as the two ricin peptides as depicted in Figure 12b and Figure 10d, respectively. The fragment ions from a tandem MS experiment of the tryptic and the synthetic peptides were matched with the sequences and compared to each other (Figure 13). The fragment ion spectra of the tryptic and the synthetic peptide were very similar and, in particular, the characteristic b3- and b4 ions gave a clear identification of the E138D variation.

To rule out incorrect localisation of the amino acid variation, four additional isomeric synthetic peptides with E135D, I137V, L139V and E146D were compared to the tryptic peptide of ricin from *R. c.* ‘Zanzibariensis’. The retention times (Figure 12e–h) and the fragment spectra (Supplementary Material Figure S3) from a tandem MS experiment of these peptides were different compared to the tryptic peptide. This yielded strong evidence that an amino acid sequence variant with E138D in the A-chain exists in ricin from *R. c.* ‘Zanzibariensis’ and *R. c.* ‘Tanzania’ but not in the other samples and cultivars examined.

#### 2.2.4. Ricin E B-Chain Amino Acid Sequence Variations

**Figure 14:**
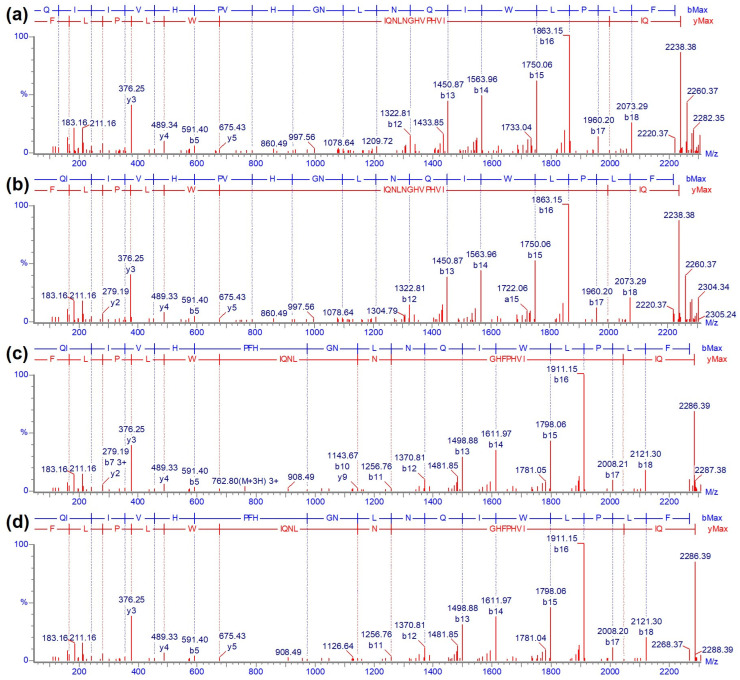
Confirmation of amino acid variation at position 250 in the B-chain of ricin employing tandem MS. Fragment ion spectra (CID) of tryptic ricin E B-chain peptide RE-T:B(244–262). (**a**) Ricin E isolated from *R. c.* ‘Carmencita’, (**b**) synthetic peptide with V250, (**c**) ricin purified from *R. c*. ‘India’ and (**d**) synthetic peptide with F250.

Ricin B-chain variants were elucidated compared to the isolated isoforms D and E from *R. c.* ‘Carmencita’ B-chains (Figure 10). *R. c*. ‘Carmencita’ ricin E, solely containing the ricin E isoform, showed a different retention time for its B-chain of 13.48 min (Figure 10d) compared to ricin E from *R. c*. ‘India’, which eluted at 13.68 min (Figure 10b), the latter showing a higher mass by 48 Da. Analysis of the tryptic peptides of the two samples revealed an amino acid variation at position 250 within peptide RE-T:B(244–262) containing either a valine (V250) or a phenylalanine (F250) in *R. c.* ‘Carmencita’ E and *R. c.* ‘India’, respectively. Peptides containing the variations were compared to their corresponding synthetic peptides by RPLC-IM-MS to confirm our data. *R. c.* ‘Carmencita’ E peptide showed nearly identical fragment ions (Figure 14a,b), retention time, molecular mass, isotopic distribution and drift time (Supplementary Material Figures S4a,b–S6a,b) to the synthetic peptide containing V250; the *R. c.* ‘India’ peptide matched with the data of the synthetic peptide containing F250 (Figure 14c,d, Supplementary Material Figures S4c,d–S6c,d). This provided strong evidence that the B-chain of ricin E exists in the two variant forms of V250 and F250, leading to a delayed elution time for ricin E B-chains comprising the phenylalanine variant (Figure 10). The remainder of the amino acid sequence of the B-chain of ricin E was in agreement with the literature [14], with exception of two amino acids at positions P70 and S71 found in reverse order (S70 and P71), which was confirmed by the cDNA [46] (Supplementary Material FASTA S1).

#### 2.2.5. Proportions of Ricin D, Ricin E (V250), Ricin E (F250) and A-Chain E138 and D138 Variants within Ricin from Different Origins

**Figure 15:**
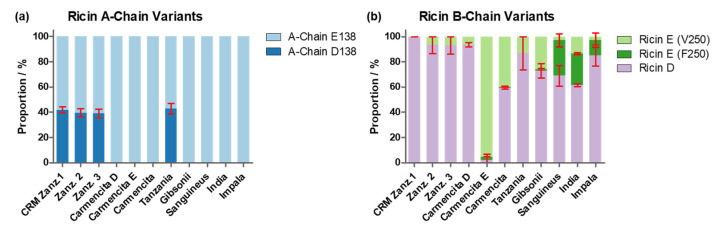
Proportions of ricin single amino acid variants within the A- and B-chains of different cultivars and isoforms compared to CRM-LS-1 (Zanz. 1). (**a**) Proportions of the E138 and D138 variants of the A-chain and (**b**) of ricin D, ricin E (V250) and ricin E (F250) B-chains. The origin of the analysed samples is indicated below the graphs. Error bars show range of data obtained from two different methods. One datapoint was obtained by measuring the tryptic peptides using RPLC-IM-MS, the other one by applying separation of B-chains with RPLC and detection by MS for the A-chain and UV (λ = 214 nm) for the B-chain variants.

The proportions of the A-chain variants (E138 and D138) and B-chains from ricin isoforms D and E, including the variants V250 and F250 of ricin E, were determined for all ricin samples from different cultivars, isolates and origins (Figure 15). The stoichiometry of E138 and D138 within the A-chain is represented in Figure 15a and summarised in Supplementary Material Table S13. Data were obtained by two different methods employing results from RPLC-MS analysis (Figure 8) and RPLC-IM-MS of the tryptic digest and extraction of peak areas of the specific peptides (R-T:A(135–166)). All three ricin samples of *R. c.* ‘Zanzibariensis’ and the sample of *R. c.* ‘Tanzania’ showed 40% of the D138 variant, whereas no D138 variant was found in the other samples.

Ricin CRM-LS-1 obtained from *R. c.* ‘Zanzibariensis’, as well as the two other samples from *R. c.* ‘Zanzibariensis’, showed a B-chain peak for ricin D, but a ricin E B-chain peak was absent (Figure 10, Supplementary Material Table S12), indicating that no significant amount of ricin E is produced in this cultivar (Figure 15b). Significant amounts of ricin E with V250 were found in ricin from *R. c.* ‘Carmencita’, *R. c.* ‘Gibsonii’ and *R. c.* ‘India’. Ricin E from *R. c.* ‘Sanguineus’ and *R. c.* ‘Impala’ contained only the F250 variant, whereas a mixture of both ricin E variants was detected in ricin from *R. c.* ‘India’ (Figure 15b). These findings were confirmed by the ratios of the tryptic peptides RD-T:B(220–236) and RE-T:B(220–236) by applying RPLC-IM-MS (Supplementary Material Table S12).

## 3. Discussion

In this work, an extensive physicochemical characterisation of the first certified ricin reference material ever produced, CRM-LS-1 (produced by the EuroBioTox project), was established. The analysis covered the detailed description of the carbohydrate and primary structure variants, and the results were compared to 10 ricin samples sourced from a total of seven different cultivars. Work conducted by Schieltz et al. [47] elucidated the content of ricin in the beans from 18 different cultivars using isotope dilution mass spectrometry, other groups determined the *Ricinus communis* source cultivar by a LC-MS method [48] or based on a complex set of biomarkers using chemometric methods [49]. Our study encompassed novel strategies discovering the molecular features of ricin purified and analysed by applying a broad method portfolio and implementing combinatorial approaches.

The four *N*-glycosylation motifs of ricin have been investigated by several groups since cytotoxicity and translocation was reported to be dependent on the glycosylation of the B-chain [10,50,51,52]. Hence, we established the glycan profile of CRM-LS-1 in detail and compared it to other samples and cultivars, resulting in 15 oligomannosidic and paucimannosidic *N*-glycan structures. While the M3X, M3FX, M5 (1,2), M6 and M7 (2) structures have previously been identified in ricin samples [7], our study described M4X (1) and M7 (1) as well as multiple low-abundance *N*-glycan structures in ricin CRM-LS-1 for the first time. M4X (2) [53] and M7 (3) [7] were described by other groups but were not identified in any of the samples analysed in this work. The glycan profile of the CRM-LS-1, covering glycopeptides and occupancies thereof, is very similar to all the cultivars analysed. Furthermore, the data for the glycan profiles was corroborated orthogonally by absolute quantification of monosaccharides and was found to be in high agreement.

The highest variation in glycosylation was reported for the second glycosylation site of the A-chain, A:N236. Its occupancy was described in the range of 25–50% [9,54,55], whereas occupancy in all of our 11 analysed samples ranged from 3% to 19%. This variation may be inherent to the source of ricin from different cultivars or may arise from the different methods applied to quantify the A-chain devoid of glycosylation at this position. Owing to the fact that the tryptic peptide covering A:N236 shared isobaric properties with a B-chain peptide, an adapted procedure needed to be developed in our study to assess occupancy accurately. Multiple *N*-glycan structures, such as M3FX [9] or oligomannosidic structures with three to six mannose residues [10], were reported at A:N236; hence, the heterogenic composition of *N*-glycan structures at this site was assumed [8]. In line with this, we also found two paucimannosidic (M3FX and M4FX) and two oligomannosidic (M5 and M6) glycan structures with varying proportions in the ricin samples from the different cultivars. The three other glycosylation sites, A:N10, B:N95 and B:N135, were occupied to >95% in all samples and cultivars investigated, consistent with earlier studies [7]. The first glycosylation site of the A-chain (A:N10) was comprised almost exclusively of M3FX. The two major glycans at the B-chain sites B:N95 and B:N135 were M6 and M7 in all our characterised samples and cultivars, which was in agreement with the findings of Kimura et al. [7], including elucidation of partially xylosylated oligomannose glycans at B:N135.

In vivo biosynthesis of ricin proceeds with expression as a single precursor polypeptide (preproricin) [56] consisting of the A-chain (preceded by a 26-mer signal peptide and a N-terminal propeptide) and the B-chain joined with a short linker propeptide [41]. After hydrolysis of the signal peptide [57], proricin is *N*-glycosylated, folded in the endoplasmic reticulum [58] and further transferred to the Golgi apparatus, where M8 *N*-glycans are trimmed [32,59] and further modified before xylosylation and fucosylation takes place [34]. Finally, proricin is passed to precursor accumulating vesicles [60] and is subsequently accumulated in storage vacuoles [61], where the nine amino acids from the N-terminal propeptide and the 11–15-amino-acid linker peptide is removed, yielding the active ricin [59]. Final trimming of plant *N*-glycans to paucimannosidic structures occurs in vacuoles [62], suggesting that the glycans found attached to the A-chain were formed in the storage vacuoles of castor bean seeds. Despite the concomitant processing and co-localisation of ricin A- and B-chains by exposure of the full-length proricin to glycan modifying golgi and vacuolar enzymes, glycan structures showed remarkable spatial differences in the two polypeptide chains and also within the two B-chain sites. While the majority of glycans at A:N10 featured paucimannosidic structures, the B-chain mainly revealed oligomannosidic carbohydrates of lower diversity, primarily showing M6 and M7 at B:N95 and substantively higher diversity with additional M4X, M5, M5X and M8 in B:N135 of all cultivars analysed. Furthermore, A-chain glycans featured xylose and fucose, whereas the *N*-glycans in the B-chain were decorated with xylose to a minor extent. Hence, B-chain glycans are stalled in an early glycan processing stage compared to the A-chain glycan forms. It remains unknown why specific glycan structures are preferentially associated with a particular polypeptide chain or with a specific glycosylation site within one protein. Given that glycosylation is not a template-driven process, it is even more surprising that this overall glycan profile is highly conserved among all the cultivars analysed herein. However, the glycan profile of agglutinin from *Sambuca nigra*, another type II ribosome-inactivating protein (RIP) with presumably conserved molecular architecture and protein folding similar to ricin upon superposition of agglutinins from *Ricinus communis* and *Abrus precatorius* with ricin (PDB 2zr1, 2q3n, 1rzo), does not build paucimannosidic glycans only confined to the A-chain. In contrast to our findings, paucimannose was present in both A- and B-chains. However, in agreement with our data, the main share of oligomannosidic carbohydrates was reported within the B-chain [63].

Polypeptide chain processing variants were detected in all samples characterised. While the N-termini of both chains were found to be identical in all ricin protein samples, we identified four different C-termini for the A-chain with a share of less than 40% corresponding to the published sequence [13,40]. Proricin is processed in storage vacuoles by the vacuolar-processing enzyme (EC: 3.4.22.-, UniProt: P49042), a specific asparaginyl endopeptidase that cleaves after the last asparagine of the propeptide to generate the N-terminus of the A-chain. The same enzyme also cleaves after the last asparagine of the linker peptide to release the A-chain with the linker peptide attached and the mature B-chain [64]. Allegedly, no processing variants were found in the N-termini of the A-chain and B-chain due to maturation by a highly specific enzyme. The four different C-termini of the A-chain, however, were presumptively the result of the maturation by a different enzyme, aspartic endopeptidase, that removed the linker peptide from the A-chain in a final step [65]. Interestingly, the shares of different C-termini detected were significantly and consistently different in ricin from *R. c.* ‘Zanzibariensis’, including the CRM-LS-1, compared to all other cultivars.

Further, amino acid sequence variants were detected within the A- and the B-chain for the first time. While initial observations of the A-chain variant E138D required A-chain mass analysis and was finally confirmed by peptide fragment analysis, the B-chain variant F250V was discovered via its different retention time in RPLC and confirmed by peptide analysis accordingly. The *R. c.* ‘Zanzibariensis’ and ‘Tanzania’ cultivars contained a roughly 40% to 60% mixture of A-chain D138 to E138, respectively, and there was 100% E138 in all other samples and cultivars used in this work. B-chain F250 was only detected in substantial amounts in *R. c.* ‘Sanguineus’, ‘India’ and ‘Impala’ cultivars but was mainly absent in the other samples. Several putative genes of the ricin gene family have been published [15] but none contained the reported A-chain variant E138D. It is conceivable that the E138D variant is absent on a genomic level in some cultivars since it was only detected in *R. c.* ‘Zanzibariensis’ and *R. c.* ‘Tanzania’. We furthermore demonstrated the coexistence of two variants, F250 and V250, of the ricin E B-chain. Interestingly, the amino acid sequence of the B-chain of ricin E was first published with valine at position 250 based on tryptic peptide analysis [14]. The cDNA encoding ricin E was sequenced only later and revealed phenylalanine at this position [46]. It is interesting to note that also the sequence of *R. c.* agglutinin, from which the C-terminus of ricin E originates, was first published with valine [66] and later cDNA analysis showed phenylalanine at this position [46,67]. The proteins were purified from seeds from several plants for each sample or cultivar; thus, it remains unclear whether the two single amino acid substitutions occurred within the same plant or if the variation reflects the result of a mixture of seeds from the different plants used.

**Figure 16:**
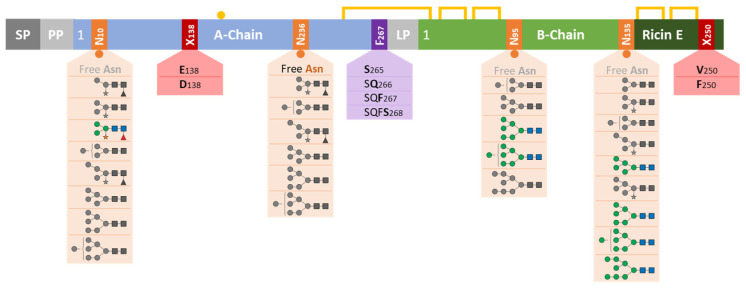
Schematic overview of *N*-glycosylations and polypeptide chain variations of ricin from *Ricinus communis,* summarising the findings from cultivars ‘Zanzibariensis’, ‘Carmencita’, ‘Tanzania’, ‘Gibsonii’, ‘Sanguineus’, ‘India’ and ‘Impala’. The figure represents preproricin with signal peptide (SP), propeptide (PP) and linker peptide (LP), which were removed during the polypeptide chain processing. The blue and green boxes symbolise the polypeptide chains, yellow lines disulphide bonds, the yellow circle a free sulfhydryl group, red boxes amino acid variations, the purple box variation in the C-terminus and orange boxes *N*-glycosylation sites. Glycans at each site are depicted and coloured according to SNFG, while structures with shares below 10% are in grey.

The four *N*-glycosylation sites with their individual composition of glycans, the two amino acid variation sites and the four C-termini of the A-chain constitute—besides the two isoforms of ricin—unique molecular signature features for ricin (Figure 16). Our study yields an extensive characterization of this protein toxin and generates an exhaustive physicochemical profile of the certified reference material CRM-LS-1 in comparison with ricin samples from other sources. Furthermore, the amino acid sequence variants may serve as a forensic attribute to identify the cultivar and hence the possible origin of ricin in biothreat scenarios.

## 4. Materials and Methods

Ricin is a highly toxic substance and listed as schedule 1 chemical by the Chemical Weapons Convention [20]. Active ricin material was therefore handled exclusively in dedicated toxin facilities (BSL-2 level) with restricted and controlled access under appropriate biosafety and biosecurity measures.

### 4.1. Materials

Synthetic peptides were obtained from GenScript Biotech Corporation and BioCat GmbH, *N*-glycan references M3 from Ludger Ltd. (Oxfordshire, UK), M5 and M6 as well as α(1–2)-mannosidase and α(1–2,3,6)-mannosidase from Agilent Technologies Inc. (Santa Clara, CA, USA). Class I α-mannosidases MNS1 and MNS3 were produced recombinantly in-house according to an adapted protocol from [32].

### 4.2. Production of Ricin Materials

To produce CRM-LS-1, an identical protocol was followed as that applied for the ricin reference material produced in the EQuATox project to obtain the maximum purity for the toxin [43]. In the present study, seeds of the *R. c.* ‘Zanzibariensis’ cultivar were used to extract ricin and to purify the toxin, while in EQuATox *R. c.* ‘Carmencita’ was used as the starting material [43]. Comprehensive characterisation of CRM-LS-1 yielded a purity of >99% and will be published in this special issue [68]. To produce the “Zanz 2”, seeds from *R. c.* ‘Zanzibariensis’ were employed. This sample was prepared along with the “Carmencita D” and the “Carmencita E” with a slightly different and shortened procedure. The mixture of ricin D and ricin E from *R. c.* ‘Carmencita’ was further processed to isolate the two isoforms by applying an anion and cation exchange chromatography step, respectively. Ricin from *R. c.* ‘Zanzibariensis’ (Zanz. 3), *R. c.* ‘Carmencita’, *R. c.* ‘Tanzania’, *R. c.* ‘Gibsonii’, *R. c.* ‘Sanguineus’, *R. c.* ‘India’ and *R. c.* ‘Impala’ was extracted similar to procedures in the literature [5] and purified using affinity and size-exclusion chromatography [69]. These samples contained a residual *Ricinus communis* agglutinin content of 2 to 10% as determined by RPLC-MS of the tryptic peptides.

### 4.3. Analysis of Hydrolytically Released Monosaccharides

For qualitative analysis and absolute quantification of the monosaccharides released from the glycans, HPAEC-PAD was used after total hydrolysis of the glycoprotein, similarly to the description by Hardy et al. [70]. For this purpose, 220 pmol ricin was precipitated with 10% (*m*/*v*) TCA for 10 min on ice. After centrifugation at 20,000× *g* for 15 min at 4 °C, the supernatant was removed and the pellet was washed with 50 µL ice-cold acetone. The suspension was repeatedly centrifuged, and the acetone supernatant was removed. The dried glycoprotein pellet was thoroughly resuspended in 100 µL 5 M TFA for qualitative analysis and in 100 µL 5 M TFA containing 1 nmol arabinose as an internal standard for quantitative analysis. The glycans were then hydrolysed for 3 h at 100 °C. Special care was taken to avoid contamination of the hydrolysis reaction with lint and dust, since glucose, arabinose and xylose are the main components of cellulose and hemicellulose and would lead to artificial signals [37]. Therefore, all samples and reagents were filtered using prerinsed Millex-GV PVDF 0.22 µm 4 mm syringe filters (Merck KGaA, Darmstadt, Germany), all vessels (e.g. cups) were rinsed with filtered water and the sample preparation was performed in a laminar flow cabinet. The hydrolysates were dried with a SpeedVac (Thermo Fisher Scientific Inc., Waltham, MA, USA), suspended in 10 µL 2-propanol and dried again. Hydrolysates were dissolved in 400 µL water prior to analysis by HPAEC-PAD and were further diluted 10 times with water. For quantification, calibrations of six calibrants were prepared in duplicate in the range of 5 nM to 160 nM for fucose, galactose, glucose and xylose and of 20 nM to 640 nM for glucosamine and mannose. An amount of 250 nM arabinose was added to each calibrant as an internal standard. HPAEC-PAD was performed using a Thermo Fisher Scientific Inc. Dionex ICS-5000+ ion chromatography system equipped with an electrochemical detector. Monosaccharides were separated with a Dionex CarboPac SA10-4 μm (2 × 50 mm precolumn and 2 × 250 mm separation column Thermo Fisher Scientific Inc.) for 5 min at 3 mM potassium hydroxide followed by a gradient to 100 mM potassium hydroxide of 0.5 min and a 2.5 min isocratic segment holding the concentration for column regeneration. After a short gradient of 0.5 min back to 3 mM potassium hydroxide, the column was re-equilibrated for 11 min. Detection was performed with a pulsed amperemeter equipped with a gold working electrode and an Ag/AgCl pH reference electrode using quadruple potential waveform (E1 = 0.1 V, t1 = 400 ms, E2 = −2.0 V, t2 = 10 ms, E3 = 0.6 V, t3 = 10 ms, E4 = −0.1 V, t4 = 60 ms).

### 4.4. Glycan Release by Hydrazinolysis

Purified ricin was precipitated at 4 °C using 10% (*m*/*v*) TCA, washed with ice-cold acetone and dried using a SpeedVac (Thermo Fisher Scientific Inc., Waltham, MA, USA). Hydrazinolysis was performed using a kit purchased from Ludger Ltd. (Oxfordshire, UK) following the manufacturer’s instructions. Briefly, the glycoprotein was incubated in hydrazine for 16 h at 85 °C. The unreacted hydrazine was removed by using a SpeedVac centrifugal evaporator equipped with a refrigerated vapor trap. After reacetylation, acidification and purification, the reducing ends of the glycans were reduced with 0.25 M sodium borohydride in 100 mM sodium borate buffer pH 12 for 10 min at 37 °C. Excess reagent was decomposed by glacial acetic acid.

### 4.5. HILIC-MS Analysis and HILIC with Peak Fractionation of Released Glycan from Ricin CRM-LS-1

An Agilent RRLC 1260 additionally equipped with a second binary pump, a valve for inline solid-phase extraction and a 6530-quadrupole time-of-flight mass spectrometer (Agilent Technologies, Inc., Santa Clara, CA, USA) was used for HILIC-MS analysis and fractionation of glycans with equal monosaccharide contents after separation on a HILIC column. Released and reduced glycans were injected into a Hypercarb precolumn (3 µm bead size, 10 × 4 mm, Thermo Fisher Scientific Inc.) for an inline solid-phase extraction and the salts were washed out with 0.1% trifluoroacetic acid and afterwards with 0.1% formic acid, both in 97% acetonitrile and 3% water at a flow of 0.25 mL/min for 4 min. To transfer the trapped glycans to the HILIC column (ACQUITY UPLC Glycoprotein BEH Amide Column, 300 Å, 1.7 µm, 2.1 mm × 50 mm, Waters Corp., Milford, MA, USA), a valve was switched to immediately change the solvent to 5% water and 0.1% formic acid in acetonitrile supplied from the second binary pump and the glycans were flushed over to the HILIC column for 2 min. After the flow was increased to 0.625 mL/min within another 2 min, a linear gradient of 30 min to 40% acetonitrile and 0.1% formic acid in water was performed to separate and elute the glycans. The detection and identification were conducted in an MS^E^ (data-independent acquisition) analysis in positive mode with alternating the collision cell voltage between 0 V and 32 V. A diode array detector (Agilent Technologies, Inc.) was used to detect peaks for the fractionation of glycans (surprisingly, glycans caused absorption peaks at a wavelength of 214 nm), and fractions were manually collected without MS detection. Fractions were dried using a SpeedVac (Thermo Fisher Scientific Inc.), dissolved in pure water and were then reinjected into the HILIC-MS for verification of the purity and analysed further with PGCC-MS.

### 4.6. PGCC-MS Analysis of Released Glycan from Ricin CRM-LS-1

The inline solid-phase extraction porous graphitised carbon chromatography-mass spectrometry method was adapted from Pabst et al. [29] and was performed on an Agilent RRLC 1260 equipped with two binary pumps coupled to a 6530-quadrupole time-of-flight mass spectrometer (Agilent Technologies, Inc.). The released and reduced glycans were trapped on a Hypercarb precolumn (3 µm bead size, 10 × 4 mm, Thermo Scientific Inc.) for an inline solid-phase extraction and the salts were washed out with 0.1% (*v*/*v*) trifluoroacetic acid. For separation, a valve was switched to couple the precolumn with a Hypercarb separation column (3 µm bead size, 100 × 2.1 mm, Thermo Fisher Scientific Inc.). Mobile phase A consisted of 10 mM NH_4_HCO_3_ and mobile phase B was 10 mM NH_4_HCO_3_ in a mixture of 60% acetonitrile and 40% water. After an initial gradient from 0% to 16.5% mobile phase B within 1 min, a gradient to 30.5% mobile phase B over 20 min was performed, followed by a 7 min gradient to 100% mobile phase B. The collision cell voltage of the QTOF was alternated between 0 V and 32 V for MSE experiments and single-stage MS mode was used for detection; relative quantification was achieved by comparing ion intensities in positive mode as recommended by Pabst et al. [29]. Therefore, the relative intensities were calculated based on the peak areas in extracted ion chromatograms (EIC) at the *m*/*z* of the monoisotopic singly and doubly charged glycan ions with a range of ±100 ppm.

### 4.7. Denaturation, Reduction and Alkylation of Ricin

Ricin in suspension from ammonium sulphate precipitated raw extract or purified ricin was precipitated at 4 °C using 10% (*m*/*v*) TCA, washed with ice-cold acetone and dried using a SpeedVac (Thermo Fisher Scientific Inc.). Pellets were solubilised in 100 µL of 6 M guanidinium hydrochloride in 50 mM Tris-HCl buffer pH 8.5 with sonification for 5 min. Samples were incubated at 56 °C for 20 min after 10 mM dithiothreitol addition. Free thiols were alkylated by the addition of 40 mM iodoacetamide and incubation at room temperature for 20 min in the dark. Quenching of unreacted iodoacetamide was performed by addition of 60 mM dithiothreitol.

### 4.8. Tryptic and Chymotryptic Digest of Ricin

Buffer exchange of denatured, reduced and alkylated ricin samples into 100 mM Tris-HCl buffer pH 8.2 containing 4 mM calcium chloride was performed using PD SpinTrap G-25 (Cytiva, Marlborough, MA, USA) following the manufacturer’s instructions. Samples were then digested at 37 °C for 100 min with mass-spectrometry-grade trypsin or for 6 h with sequencing-grade chymotrypsin (Promega Corp., Fitchburg, WI, USA) with an enzyme-to-protein ratios of 1:62 and 1:31, respectively. The enzymic reaction was quenched by adding 1% acetic acid.

### 4.9. HILIC-IM-MS Analysis of Tryptic and Chymotryptic Peptides

HILIC-IM-MS of glycopeptides was performed on an ACQUITY UPLC I-Class PLUS with a SYNAPT G2-Si (Waters Corp.). Aqueous peptide samples were diluted to 50% acetonitrile and 1% dimethyl sulfoxide prior to injection on an ACQUITY Premier Glycan BEH Amide, 2.1 × 150 mm HILIC column (Waters Corp.). After an initial isocratic segment of 1 min separation with 14.2% water and 0.1% formic acid in acetonitrile, a gradient to 51% water with 0.1% formic acid in 25 min, followed by a steeper gradient to 97% water and 0.1% formic acid within 0.5 min was conducted maintaining a flow of 0.25 mL/min. A HDMSE in positive-resolution mode was used with a voltage ramp from 20 V to 45 V in the transfer region to measure mass and fragment spectra. Peptide identification and determination of relative shares of the glycopeptides were conducted using BiopharmaLynx™ (Waters Corp.). Peak areas of C-terminal peptides of the ricin A-chain with different C-termini were measured in extracted ion chromatograms of the singly and doubly charged ions using MassLynx (Waters Corp.).

### 4.10. RPLC-IM-MS Analysis of Tryptic Peptides

Peptides were analysed using the RPLC-IM-MS method on an ACQUITY UPLC I-Class PLUS with a SYNAPT G2-Si (Waters Corp.) with an ACQUITY UPLC Peptide BEH C18, 2.1 × 50 mm column (Waters Corp.). The gradient concentrations were mixed with 3% acetonitrile and 0.1% formic acid in water as eluent A and 5% water and 0.1% formic acid in acetonitrile as eluent B. The flow was set to 0.25 mL/min and column was heated to 60 °C. After an initial isocratic segment with 100% eluent A for 1 min, a linear gradient to 40% eluent B in 23.5 min and then a steeper gradient to 100% eluent B in 2 min was applied. A shorter segmented gradient was used for analysis of the amino acid sequence variants in the peptide R-T:A(135–166) of the A-chain and its variants. Eluent B was increased from 0 to 30% in the first minute of the gradient followed by a flat gradient to 40% in 9.5 min and then to 100% eluent B in 2 min. The mass spectrometer was set to the positive-resolution mode with ion mobility separation enabled and the calibrated *m*/*z* range from 50 to 2000 was used. Fragment spectra were acquired with a voltage ramp from 20 V to 45 V in the transfer region. BiopharmaLynx™ (Waters Corp.) was used for qualitative and quantitative evaluation.

### 4.11. RPLC-MS Analysis of the Intact A- and B-Chains of Ricin CRM-LS-1

Denatured, reduced and alkylated ricin was used for the RPLC-MS measurements. Analysis of intact A- and B-chains of ricin CRM-LS-1 was performed three times on an Agilent RRLC 1260 equipped with a ACQUITY UPLC Protein BEH C4, 2.1 × 50 mm column (Waters Corp.) coupled with a 6530-quadrupole time-of-flight mass spectrometer (Agilent Technologies, Inc.). Eluent A consisted of 7% 2-propanol, 3% acetonitrile, 3% butanol, 0.2% formic acid and 0.03% trifluoracetic acid in water and eluent B was 30% acetonitrile, 10% water, 3% butanol, 0.25% formic acid and 0.01% trifluoroacetic acid in 2-propanol. The flow was set to 0.6 mL/min with 100% eluent A and the column was heated to 90 °C. After an initial gradient to 3% B in 1 min, a separation gradient to 18% within 21 min was performed followed by a steeper gradient to 75% in 2 min. The mass spectrometer was set to positive mode with a *m*/*z* range of 3200 and a fragmentor voltage of 200 V. Drying gas was 12 L/min at 360 °C with a nebuliser pressure of 40 psig and sheath gas was 8 L/min at 355 °C. Mass spectra were deconvoluted with MassHunter Bioconfirm (Agilent Technology, Inc.) maximum entropy algorithm and proportion of mass peaks to determine the occupancy of A:N236 were calculated using DAR Calculator (Agilent Technology, Inc.).

### 4.12. Measurement of Occupancy of A:N236 by RPLC-MS of Lys-C Digested A-Chain

The occupancy of A:N236 of ricin samples from different origins including a fourth measurement of the ricin CRM-LS-1 was determined side-by-side for comparison. The samples were digested with Lys-C to reduce the complexity of mass spectra of the ricin A-chain caused by four different C-termini. For this purpose, denatured, reduced and alkylated samples were diluted to 0.62 M guanidinium hydrochloride with 100 mM Tris-HCl buffer, pH 8.2 containing 4 mM calcium chloride and 100 mM lactose and 0.75 g/L glutathione (oxidised). Mass-spectrometry-grade recombinant Lys-C (Promega Corp.) was added in an enzyme-to-protein ratio of 1:37.5 and incubated at 37 °C for 16 h. The RPLC-MS measurements were performed on an ACQUITY UPLC I-Class PLUS with a SYNAPT G2-Si described later and the relative shares were determined using the peak areas of the singly and doubly glycosylated A-chain in extracted ion chromatograms of the [M + 17H]^+17^ ions.

### 4.13. RPLC-MS Analysis of the Intact A- and B-Chains of Ricin Using ACQUITY UPLC I-Class PLUS with a SYNAPT G2-Si

An ACQUITY UPLC I-Class PLUS with a SYNAPT G2-Si (Waters Corp.) was used to measure the intact ricin A- and B-chains of all ricin samples in a side-by-side comparison and Lys-C digested A-chain to measure the occupancy of A:N236. Eluent A was 3% acetonitrile, 0.1% formic acid and 0.03% trifluoroacetic acid in water and eluent B was 5% water, 0.1% formic acid and 0.03% trifluoro acetic acid in acetonitrile. A BioResolve RP mAb Polyphenyl Column (2.1 mm × 150 mm) (Waters Corp.) was used at 90 °C for the separation. The initial concentration of 5% eluent B was increased in 1 min to 15%, then to 51% in 20 min in linear gradients and finally to 100% in 2.25 min in a convex gradient (8) maintaining a flow of 0.2 mL/min. The mass spectrometer was used in positive-resolution mode with a calibrated *m*/*z* range from 500 to 5000. The deconvolution of protein spectra was performed with MassLynx MaxEnt 1 algorithm (Waters Corp.).

### 4.14. Quantification of Ricin D, Ricin E and Agglutinin

Ricin D (RD) and E (RE) feature identical A-chains and the N-terminal part of the B-chain, whereas ricin E and agglutinin (RCA) have identical C-terminal sequences of the B-chain. Hence, the first step to determine the ratio of ricin D and E was to measure the agglutinin content. The signals of the tryptic peptides from the common ricin D and E (R) A-chain R-T:A(49–56) and from the agglutinin A-chain RCA-T:A(49–56) were measured. The sequence of these peptides differs only in one amino acid and thus the sensitivity in RPLC-MS analysis was presumed to be equal. Thus, the ratio of the signals reflected the proportion of agglutinin in the ricin samples. The unique B-chain peptide RD-T:B(220–236) was used to measure the signal of ricin D. Ricin E and agglutinin shared the identical peptides RE-T:B(220–236) and RCA-T:B(220–236), which were very similar (one amino acid exchanged) to the RD-T:B(220–236) of ricin D. The ratio of ricin D to the sum of ricin E and agglutinin was calculated with these peptides. Finally, the proportion of ricin E was calculated based on the share of ricin D and agglutinin.

## Figures and Tables

**Table 1:** *N*-Glycans released from ricin CRM-LS-1 analysed by PGCC-MS. The proportions with standard deviations (SD) were calculated from the peak area (A) of singly and doubly charged ions in the extracted ion chromatograms.

Label	Structure	[M + H]^+^	[M + 2H]^2+^	Retention t/min	Proportion A/%	SD s/%
M2FX	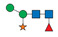	1029	515	8.1	0.5	0.15
M3	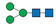	913	457	11.9	1.1	0.13
M3X	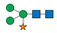	1045	523	12.4	1.9	1.00
M3FX	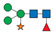	1191	596	8.8	25.0	5.50
M4 (3)	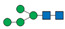	1075	538	8.5	0.4	0.05
M4 (1)	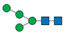	1075	538	11	1.0	0.48
M4X	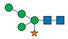	1207	604	11.9	3.5	1.30
M4FX	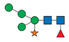	1354	677	8.4	1.6	0.80
M5 (1,3)	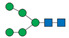	1237	619	8.3	0.6	0.05
M5 (1,2)	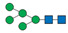	1237	619	13.6	11.3	0.47
M5X	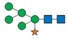	1369	685	14.5	1.2	0.15
M6	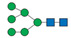	1400	700	9.9	35.2	1.30
M7 (1)	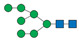	1562	781	9.5	2.5	0.30
M7 (2)	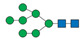	1562	781	9.9	10.0	1.99
M8	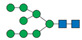	1724	862	9.6	4.1	0.52

**Table 2:** Proportions (peak area, A/%) and standard deviations (s/%) of glycans in ricin CRM-LS-1 at the four glycosylation motifs (A:N10, A:236, B:N95 and B:N135) measured by HILIC-IM-MS from four independent sample preparations. Isomeric glycan structures were not resolved; therefore, the sums of shares are stated.

Glycan	A:N10		A:N236		B:N95		B:N135	
	A/%	s/%	A/%	s/%	A/%	s/%	A/%	s/%
Free N	0.5	0.3	87	2.3	1.7	0.8	0.2 ^#^	
M2FX	1.5	1.5						
M3							0.2	0.0
M3X	1.5	1.9					0.2	0.1
M3FX	89.5	5.2	1.9	0.9				
M4	0.5 ^#^		0.5 ^#^		0.7	0.1	1.4	0.2
M4X							7.8	0.9
M4FX	1.0	0.7	3.0	0.1	0.1 ^#^			
M5	5.4	5.2	3.7	0.6	1.1	0.2	15.8	0.5
M5X							3.4	0.4
M6	0.3	0.1	2.3	0.4	77.5	1.1	22.9	1.6
M7	0.5 ^#^		2.4	0.9	19.0	2.0	28.5	1.3
M8					0.7	0.5	19.9	1.7

^#^ Only one of the four measurements showed detectable signals.

## Data Availability

Data presented in this study are available upon request to the corresponding author, if compliant with the security policy.

## Supplementary Materials

The following supporting information can be downloaded at: https://www.mdpi.com/article/10.3390/toxins16060243/s1, Figure S1: Proportions of N-glycans of ricin CRM-LS-1 using PGCC-MS of released glycans (blue) and HILIC-IM-MS of glycopeptides (green), Figure S2: Fragment spectra (CID) from tryptic peptide R-T:A(135–166) with variation at position 138 revealing either a glutamate or an aspartate residue. Figure S3: Fragment spectra (CID) of synthetic peptides R-T:A(135–166) with amino acid exchanges, Figure S4: Extracted ion chromatograms of triple charged peptides RE-T:B(244–262) of ricin E, B-chain with V250 and F250, Figure S5: Mass spectra of the tryptic peptide RE-T:B(244–262) of B-chain of ricin E which occurs with V250 and F250. Figure S6: Arrival time distributions of tryptic peptide RE-T:B(244–262) of B-chain of ricin E with V250 or F250, Table S1: Tryptic peptides of fraction of B-chain peak in RPLC of reduced and alkylated ricin CRM-LS-1 analysed by RPLC-MS, Table S2: Tryptic peptides of fraction of A-chain peak in RPLC of reduced and alkylated ricin CRM-LS-1 analysed by RPLC-MS, Table S3: C-terminal peptides RD-T:A(259–C-terminus) of ricin A-chain with four varying C-termini detected in RPLC-HDMSE, Table S4: C-terminal peptides RD-T:A(259–C-terminus) of ricin A-chain with four varying C-termini detected in HILIC-HDMSE, Table S5: Monosaccharide content of ricin CRM-LS-1 as absolute concentrations and in a monosaccharide to protein ratios with standard deviation (s), Table S6: Proportions in % of tryptic peptide RD-T:A(5–26) glycosylated at RD-A:N10 with the glycan in the column header measured using HILIC-HDMSE. Table S7: Proportions in % of tryptic peptide RD-T:A(236–239) glycosylated at RD-A:N236 with the glycan in the column header measured using HILIC-HDMSE, Table S8: Proportions in % of tryptic peptide RD-T:B(90–102) glycosylated at RD-B:N95 with the glycan in the column header measured using HILIC-HDMSE. Table S9: Proportions in % of chymotryptic peptide RD-C:B(132–140) glycosylated at RD-B:N135 with the glycan in the column header measured using HILIC-HDMSE, Table S10. Duplicates of proportions in % of different A-chain C-termini measured by LC-MS, Table S11: Proportions in % of different A-chain C-termini measured by HILIC-IMS-MS, Table S12: Proportions in % of isoforms in ricin D and the two amino acid variants (V250 and F250) of ricin E, Table S13: Proportions in % of two amino acid variants (E138 and D138) of the A-chain of ricin, FASTA S1: Amino acid sequences of ricin used in this article. A-chain of ricin D and E were identical.
