# Supplementary material for: Glycan Profile and Sequence Variants of Certified Ricin Reference Material and Other Ricin Samples Yield Unique Molecular Signature Features
Source: Toxins (Basel). 2024 May 26;16(6):243. doi: 10.3390/toxins16060243 (PMC11209631; doi:10.3390/toxins16060243)
Supplement: Supplementary file 1 [file toxins-16-00243-s001.zip › toxins-2974564-supplementary.pdf]

toxins

MDPI

Supplementary Data:

Glycan Profile and Sequence Variants of Certified Ricin Reference Material and other Ricin Samples Yield Unique Molecular Signature Features

Roland Josuran [1], Andreas Wenger [1], Christian Müller [2], Bettina Kampa [3], Sylvia Worbs [3], Brigitte G. Dorner [3], and Sabina Gerber [1,*]

[1] Institute of Chemistry and Biotechnology, Zurich University of Applied Sciences ZHAW, 8820 Wädenswil, Switzerland
[2] Spiez Laboratory, Federal Office for Civil Protection, 3700 Spiez, Switzerland
[3] Biological Toxins (ZBS3), Centre for Biological Threats and Special Pathogens, Robert Koch Institute, 13353 Berlin, Germany
* Correspondence: sabina.gerber@zhaw.ch; Tel.: +41-58-934-54-28

1. Comparison of glycan proportions of released glycans and glycopeptides

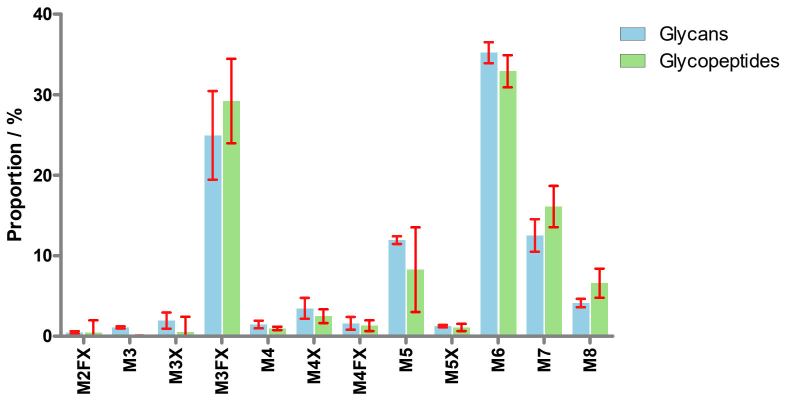

Figure S1. Proportions of *N*-glycans of ricin CRM-LS-1 using PGCC-MS of released glycans (blue) and HILIC-IM-MS of glycopeptides (green). Error bars represents the standard deviation of replicate analysis of released glycans performed from two operators on different days in triplicates from different vials and from four independent sample preparations for the glycopeptide analysis.

2. Identification of peaks in RPLC-MS of reduced and alkylated ricin CRM-LS-1

Table S1. Tryptic peptides of fraction of B-chain peak in RPLC of reduced and alkylated ricin CRM-LS-1 analysed by RPLC-MS.

Label	Sequence	t_R / min	Mass / Da
B(1–12)	ADVCMDPEPIVR	18.85	1400.6554
B(17–24)	NGLCVDVR	13.00	931.4627
B(28–40)	FHNGNAIQLWPCK	19.96	1583.7808
B(41–52)	SNTDANQLWTLK	18.75	1389.7023
B(63–89)	CLTTYGYSPGVYVMIYDCNTAATDATR	26.83	3062.3535
B(169–187)	AEQQWALYADGSIRPQQNR	18.93	2230.1105
B(188–198)	DNCLTSDSNIR	6.01	1293.5633
B(188–203)	DNCLTSDSNIRETVVK	16.21	1849.9065
B(204–215)	ILSCGPASSGQR	3.19	1231.5990
B(216–219)	WMFK	12.71	610.2982
B(220–236)	NDGTILNLYSGLVLDVR	31.96	1861.0055

Table S2. Tryptic peptides of fraction of A-chain peak in RPLC of reduced and alkylated ricin CRM-LS-1 analysed by RPLC-MS.

Label	Sequence	t_R / min	Mass / Da
A(5–26)	QYPIINFTTAGATVQSYTNFIR	30.57	3674.706
A(32–39)	LTTGADVR	1.58	831.4473
A(40–48)	HEIPVLPNR	15.40	1073.6110
A(49–56)	VGLPINQR	15.05	895.5340
A(86–114)	AGNSAYFFHPDNQEDAEAITHLFTDVQNR	28.29	3306.5091
A(115–125)	YTFAFGGNYDR	18.18	1309.5861
A(126–134)	LEQLAGNLR	13.77	1012.5786
A(198–213)	SAPDPSVITLENSWGR	23.10	1727.8552
A(214–234)	LSTAIQESNQGAFASPIQLQR	23.50	2258.1769

Table S3. C-terminal peptides RD-T:A(259–C-terminus) of ricin A-chain with four varying C-termini detected in RPLC-HDMSE.

Sequence	t_R / min	Drift / bins	m/z	Fragments
CAPPPSS	1.3	95	715.3109	b3;y3;y4;y5
CAPPPSSQ	1.3	111	843.3718	b3;b7;y6
CAPPPSSQF[1]	6.2	136	495.7239	b2;b3;b4;b5;b6;b7;b8;y1;y5;y6;y7
CAPPPSSQFS	5.6	150	1077.4747	b1;b2;b4;b6;b7;b8;b9;y2;y4;y6;y8

Table S4. C-terminal peptides RD-T:A(259–C-terminus) of ricin A-chain with four varying C-termini detected in HILIC-HDMSE.

Sequence	t_R / min	Drift / bins	m/z	Fragments
CAPPPSS	6.7	94	715.3113	b2;b3;b4;y4;y5
CAPPPSSQ	7.9	112	843.3728	y5;y6
CAPPPSSQF[1]	6.2	135	990.4415	b2;b3;b4;b8;y6;y7
CAPPPSSQFS	7.2	148	1077.475	b2;b4;b8;b9;y2;y8

[1] Published C-terminus of A-chain.

Table S5. Monosaccharide content of ricin CRM-LS-1 as absolute concentrations and in a monosaccharide to protein ratios with standard deviation (s). Values in "monosaccharide" columns were measured with HPAEC-PAD of hydrolysed samples by two operators on a different day in sixfold setup (operator 1) and in triplicate setup (operator 2) from a different vial each operator. Values in "glycopeptide" columns were calculated from the proportions of glycans at the four glycosylation sites acquired by glycopeptide analysis with HILIC-MS from three independent sample preparations.

	**Monosaccharides**				**Glycopeptides**	
	c / µM	s / µM	Ratio	s	Ratio	s
Fuc	17	2.4	1.0	0.09	0.9	0.04
Gal	0.0	n.a.	0.0	n.a.	0.0	n.a.
GlcN	90	3.5	5.4	0.50	6.2	0.04
Glc	1	1.9	0.08	0.11	0.0	n.a.
Xyl	16	1.6	0.95	0.04	1.1	0.07
Man	256	7.3	15.4	0.38	16.2	0.06

3. Glycosylation of ricin D and E isolated from different cultivars

Table S6. Proportions in % of tryptic peptide RD-T:A(5–26) glycosylated at RD-A:N10 with the glycan in the column header measured using HILIC-HDMSE.

**Sample**	Free N	M2FX	M3X	M3FX	M4FX	M5
Zanzibariensis 1	0.16	1.27	0.87	96.5	0.48	0.72
Zanzibariensis 2	0	0.57	0	99.1	0.15	0.22
Zanzibariensis 2	0.04	0.28	0	99.4	0.00	0.31
Carmencita D	0.67	0.13	0	98.4	0.63	0.19
Carmencita E	3.43	0.52	0	96.0	0	0.00
Carmencita	0.70	0.26	0	98.1	0.67	0.27
Tanzania	0.12	0.07	0.23	98.8	0.36	0.42
Gibsonii	0.70	0.00	0	98.9	0.24	0.18
Sanguineus	0.61	0.31	0	97.6	1.14	0.36
India	0.11	0.18	0	99.4	0.30	0
Impala	1.06	0.38	0	96.9	1.30	0.38

Table S7. Proportions in % of tryptic peptide RD-T:A(236–239) glycosylated at RD-A:N236 with the glycan in the column header measured using HILIC-HDMSE.

**Sample**	Free N[1]	M3FX	M4FX	M5	M6
Zanzibariensis 1	86.4	3.3	3.1	2.8	2.7
Zanzibariensis 2	85.9	3.6	3.8	1.9	2.6
Zanzibariensis 2	84.0	4.1	4.3	2.2	1.9
Carmencita D	86.3	3.7	4.0	1.5	1.6
Carmencita E	87.0	3.2	4.2	1.7	1.1
Carmencita	84.7	2.9	5.1	1.8	1.5
Tanzania	85.4	2.2	3.3	2.2	2.1
Gibsonii	87.2	3.1	5.0	2.6	0
Sanguineus	85.7	2.2	4.5	4.0	0.9
India	80.9	6.0	7.1	1.4	1.4
Impala	96.9	0.6	1.0	0.9	0.2

[1] The proportion of non-glycosylated RD-A:N236 was assessed using the Lys-C digested A-chain analysed by RPLC-MS.

Table S8. Proportions in % of tryptic peptide RD-T:B(90–102) glycosylated at RD-B:N95 with the glycan in the column header measured using HILIC-HDMSE.

**Sample**	Free N	M4	M5	M6	M7	M8
Zanzibariensis 1	0.85	0.75	1.30	77.5	19.4	0.17
Zanzibariensis 2	0.38	0.14	0.79	77.5	21.0	0.14
Zanzibariensis 2	0.52	0.18	0.92	77.0	21.2	0.15
Carmencita D	0.72	2.05	1.04	84.2	11.9	0.11
Carmencita E	1.75	3.01	1.10	87.1	7.0	0.07
Carmencita	0.55	0.74	1.39	85.0	12.2	0.11
Tanzania	0.87	0.56	0.83	74.9	22.6	0.31
Gibsonii	0.82	0.30	0.90	87.4	10.6	0
Sanguineus	1.20	0.83	1.24	85.7	11.0	0
India	0.53	0.22	0.83	85.0	13.4	0
Impala	1.92	0.86	1.11	86.9	9.2	0

Table S9. Proportions in % of chymotryptic peptide RD-C:B(132–140) glycosylated at RD-B:N135 with the glycan in the column header measured using HILIC-HDMSE.

**Sample**	M4	M4X	M5	M5X	M6	M7	M8
Zanzibariensis 1	1.3	6.6	16.0	3.0	25.3	30.5	17.4
Zanzibariensis 2	1.4	7.6	14.5	3.3	26.4	33.2	13.5
Zanzibariensis 2	1.3	7.2	14.9	3.2	26.5	34.2	12.7
Carmencita D	1.4	10.0	13.7	4.4	27.3	29.7	13.5
Carmencita E	2.1	14.7	15.3	5.6	25.1	25.6	11.6
Carmencita	1.6	11.5	15.7	6.4	24.4	28.8	11.5
Tanzania	0.8	4.6	12.3	2.1	29.8	36.7	13.7
Gibsonii	0.9	6.7	19.6	4.9	30.0	31.4	6.5
Sanguineus	1.0	6.6	23.9	3.9	26.4	27.3	10.8
India	1.5	13.8	14.6	5.9	27.8	29.7	6.9
Impala	1.3	7.8	26.5	3.5	25.2	25.1	10.5

4. Variation of ricin A-chain C-terminus detected in different cultivars

Table S10. Duplicates of proportions in % of different A-chain C-termini measured by LC-MS.

**Sample**	CAPPPSS		CAPPPSSQ		CAPPPSSQF[1]		CAPPPSSQFS	
Zanzibariensis 1	26.1	27.6	13.4	14.4	31.7	28.3	28.9	29.7
Zanzibariensis 2	23.1	23.9	13.7	15.1	36.1	33.0	27.2	27.9
Zanzibariensis 2	23.5	24.1	14.7	15.8	36.7	33.9	25.1	26.3
Carmencita D	21.8	22.1	7.4	7.4	19.2	17.0	51.6	53.4
Carmencita E	22.9	23.2	7.9	8.1	21.2	18.6	48.1	50.0
Carmencita	27.2	26.6	9.2	11.8	19.3	18.9	44.4	42.6
Tanzania	23.8	24.5	7.6	9.4	22.6	20.2	45.9	45.9
Gibsonii	20.6	21.1	10.8	11.6	23.2	20.9	45.5	46.4
Sanguineus	21.4	21.8	7.7	9.7	19.5	16.9	51.4	51.6
India	17.7	18.7	11.1	12.2	31.3	28.6	39.8	40.5
Impala	19.9	20.3	8.8	11.0	19.9	18.7	51.4	50.0

[1] Published C-terminus of A-chain.

Table S11. Proportions in % of different A-chain C-termini measured by HILIC-IMS-MS.

**Sample**	CAPPPSS	CAPPPSSQ	CAPPPSSQF[1]	CAPPPSSQFS
Zanzibariensis 1	28.4	6.2	35.3	30.0
Zanzibariensis 2	25.2	5.3	42.7	26.8
Zanzibariensis 2	25.5	6.2	42.7	25.5
Carmencita D	25.8	2.9	18.1	53.2
Carmencita E	21.6	2.8	22.7	52.9
Carmencita	27.7	6.2	20.0	46.2
Tanzania	25.1	2.7	22.2	50.1
Gibsonii	20.7	6.1	26.6	46.6
Sanguineus	22.5	4.1	19.2	54.2
India	16.1	5.6	33.4	44.9
Impala	20.8	5.4	21.3	52.6

[1] Published C-terminus of A-chain.

5. Ricin sequence variants

5.1. Ricin A-chain variants (E138/D138)

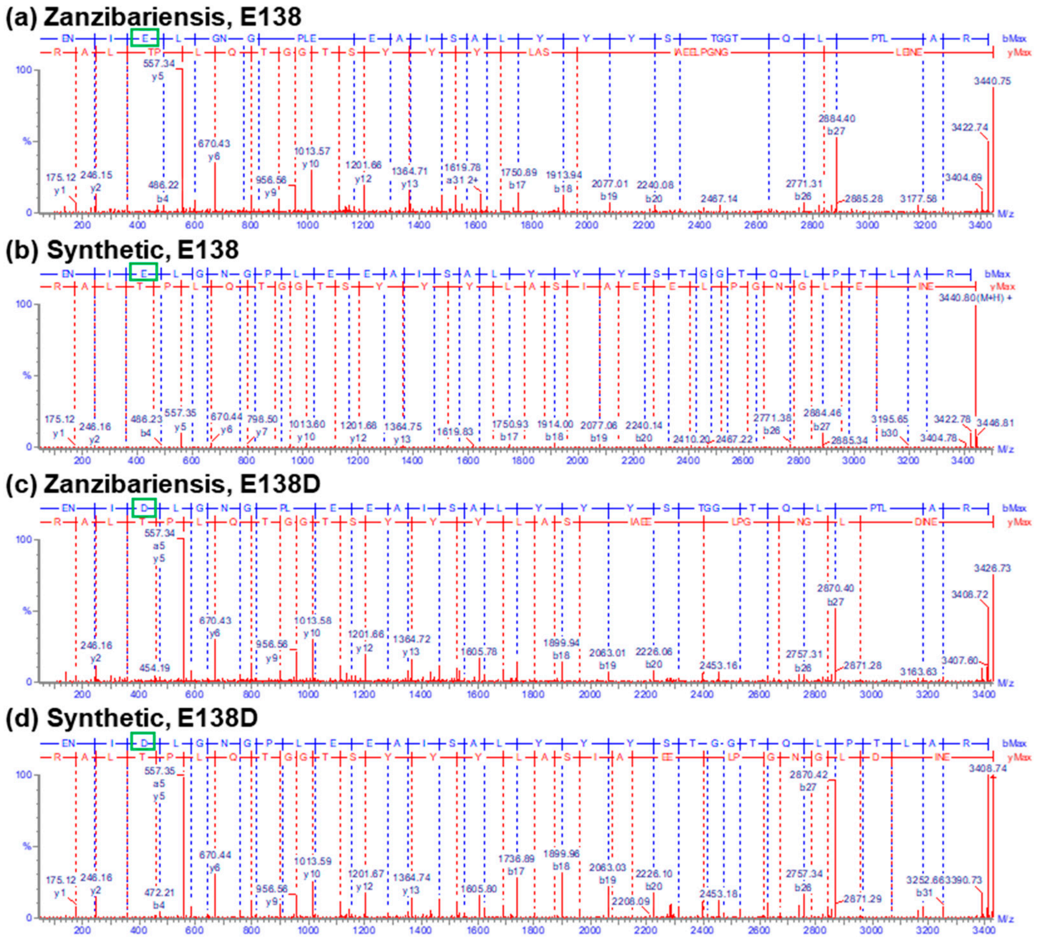

Figure S2. Fragment spectra (CID) from tryptic peptide R-T:A(135–166) with variation at position 138 revealing either a glutamate or an aspartate residue. (**a** and **b**) Show the spectra of tryptic peptide of ricin purified from *R. c.* Zanzibariensis with the sequence $_{135}$ENIELGNGPLEEAISALYYYSTGGTQLPTLAR^{166} compared to fragment ions of the corresponding synthetic peptide, respectively and (**c** and **d**) show the spectra of tryptic peptide of ricin purified from *R. c.* Zanzibariensis with the sequence $_{135}$ENIDLGNGPLEEAISALYYYSTGGTQLPTLAR^{166} compared to fragment ions of the corresponding synthetic peptide, respectively.

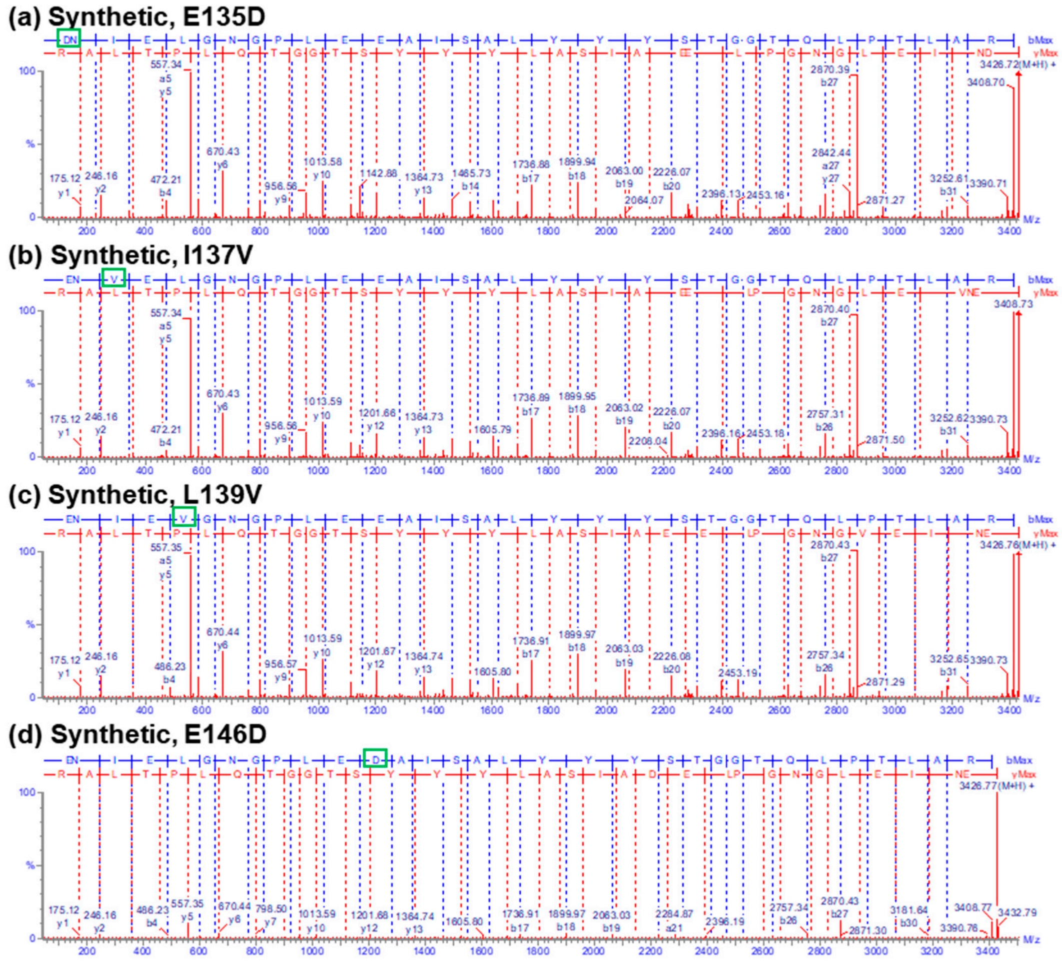

Figure S3. Fragment spectra (CID) of synthetic peptides R-T:A(135–166) with amino acid exchanges as indicated in the header.

5.1. Ricin E B-Chain variants (V250/F250)

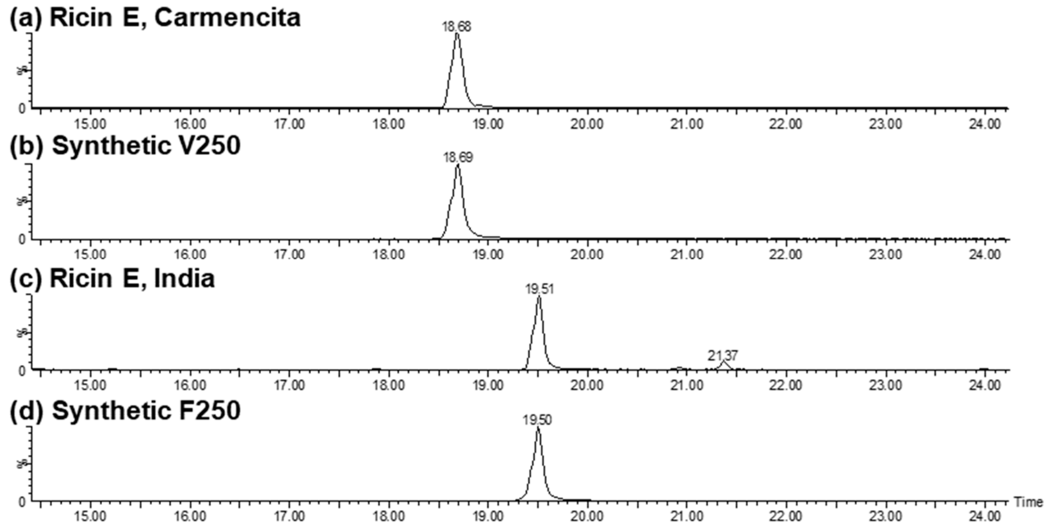

Figure S4: Extracted ion chromatograms of triple charged peptides RE-T:B(244–262) of ricin E, B-chain with V250 in **a**) and **b**) and F250 in **c**) and **d**). Tryptic peptides from ricin E isolated from *R. c.* Carmencita in **a**), and from *R. c.* India in **c**). Synthetic peptides with V250 and F250 in panel **b**) and **d**), respectively.

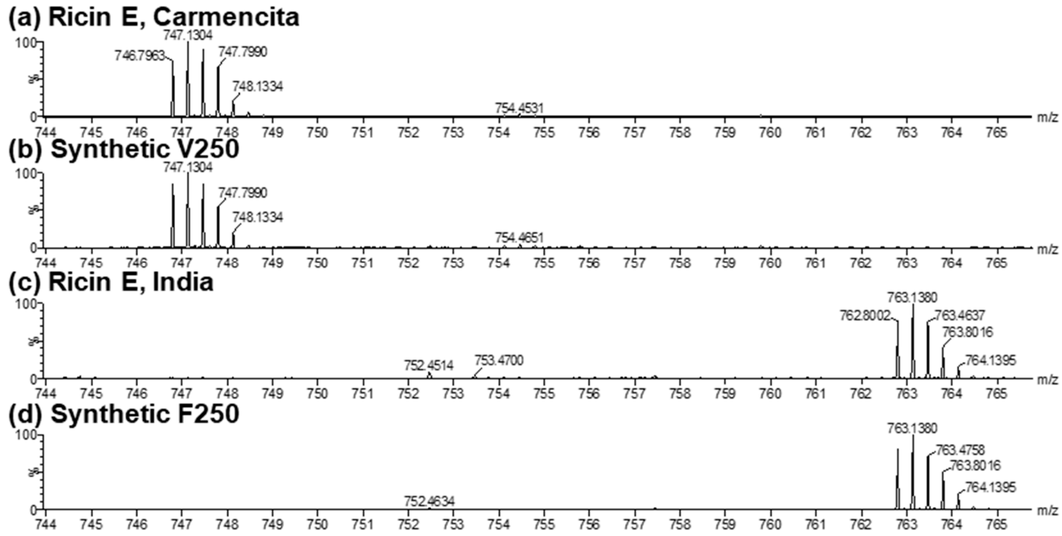

Figure S5: Mass spectra of the tryptic peptide RE-T:B(244–262) of B-chain of ricin E which occurs with V250 and F250. **a**) Tryptic peptide of ricin E isolated from *R. c.* Carmencita, **b**) synthetic peptide with V250, **c**) tryptic peptide of ricin purified from *R. c.* India, **d**) synthetic peptide with F250.

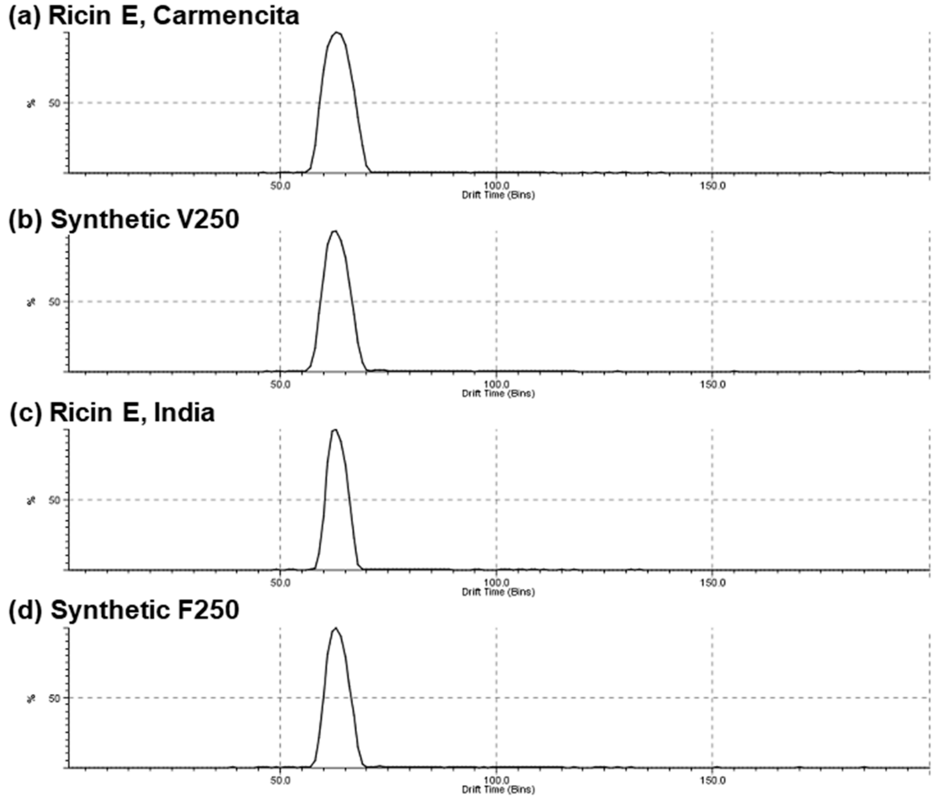

Figure S6: Arrival time distributions of tryptic peptide RE-T:B(244–262) of B-chain of ricin E with V250 or F250. **a**) Tryptic peptide of ricin E isolated from *R. c.* Carmencita, **b**) synthetic peptide with V250, **c**) tryptic peptide of ricin purified from *R. c.* India, **d**) synthetic peptide with F250.

5.2. *Ricin Amino Acid Sequences*

FASTA S1. Amino acid sequences of ricin used in this article. A-chain of ricin D and E were identical.

```
>Ricin D A chain|E138|P02879|36-302
IFPKQYPIINFTTAGATVQSYTNFIRAVRGRLTTGADVRHEIPVLPNRVGLPINQRFILV
ELSNHAELSVTLALDVTNAYVVGYRAGNSAYFFHPDNQEDAEAITHLFTDVQNRYTFAFG
GNYDRLEQLAGNLRENIELGNGPLEEAISALYYYSTGGTQLPTLARSFIICIQMISEAAR
FQYIEGEMRTRIRYNRRSAPDPSVITLENSWGRLSTAIQESNQGAFASPIQLQRRNGSKF
SVYDVSILIPIIALMVYRCAPPPSSQF

>Ricin D A chain|D138|P02879|36-302
IFPKQYPIINFTTAGATVQSYTNFIRAVRGRLTTGADVRHEIPVLPNRVGLPINQRFILV
ELSNHAELSVTLALDVTNAYVVGYRAGNSAYFFHPDNQEDAEAITHLFTDVQNRYTFAFG
GNYDRLEQLAGNLRENIDLGNGPLEEAISALYYYSTGGTQLPTLARSFIICIQMISEAAR
FQYIEGEMRTRIRYNRRSAPDPSVITLENSWGRLSTAIQESNQGAFASPIQLQRRNGSKF
SVYDVSILIPIIALMVYRCAPPPSSQF

>Ricin D B chain|P02879|315-576
ADVCMDPEPIVRIVGRNGLCVDVRDGRFHNGNAIQLWPCKSNTDANQLWTLKRDNTIRSN
GKCLTTYGYSPGVYVMIYDCNTAATDATRWQIWDNGTIINPRSSLVLAATSGNSGTTLTV
QTNIYAVSQGWLPTNNTQPFVTTIVGLYGLCLQANSGQVWIEDCSSEKAEQQWALYADGS
IRPQQNRDNCLTSDSNIRETVVKILSCGPASSGQRWMFKNDGTILNLYSGLVLDVRASDP
SLKQIILYPLHGDPNQIWLPLF

>Ricin E B chain|V250|315-576
ADVCMDPEPIVRIVGRNGLCVDVRDGRFHNGNAIQLWPCKSNTDANQLWTLKRDNTIRSN
GKCLTTYGYSPGVYVMIYDCNTAATDATRWQIWDNGTIINPRSSLVLAATSGNSGTTLTV
QTNIYAVSQGWLPTNNTQPFVTTIVGLYGMCLQANSGKVWLEDCTSEKAEQQWALYADGS
IRPQQNRDNCLTTDANIKGTVVKILSCGPASSGQRWMFKNDGTILNLYNGLVLDVRRSDP
SLKQIIVHPVHGNLNQIWLPLF

>Ricin E B chain|F250|315-576
ADVCMDPEPIVRIVGRNGLCVDVRDGRFHNGNAIQLWPCKSNTDANQLWTLKRDNTIRSN
GKCLTTYGYSPGVYVMIYDCNTAATDATRWQIWDNGTIINPRSSLVLAATSGNSGTTLTV
QTNIYAVSQGWLPTNNTQPFVTTIVGLYGMCLQANSGKVWLEDCTSEKAEQQWALYADGS
IRPQQNRDNCLTTDANIKGTVVKILSCGPASSGQRWMFKNDGTILNLYNGLVLDVRRSDP
SLKQIIVHPFHGNLNQIWLPLF
```

6. Ricin E Isoforms and Polymorphisms in different cultivars

Table S12. Proportions in % of isoforms in ricin D and the two amino acid variants (V250 and F250) of ricin E. Columns labelled peptides show data obtained by RPLC-HDMSE of tryptic peptide B(220–236). Columns labelled with B-chain were obtained with RPLC-UV of intact reduced and alkylated subunits of ricin. Values below the lower limit of detection were indicated as < LOD.

**Sample**	**Ricin E (V250)**		**Ricin E (F250)**		**Ricin D**	
	Peptides	B-Chain	Peptides	B-Chain	Peptides	B-Chain
Zanzibariensis 1	0.1	< LOD	< LOD	< LOD	99.9	100.0
Zanzibariensis 2	13.6	< LOD	< LOD	< LOD	86.4	100.0
Zanzibariensis 2	14.2	< LOD	< LOD	< LOD	85.8	100.0
Carmencita D	4.7	8.3	< LOD	< LOD	95.3	91.7
Carmencita E	95.2	95.6	< LOD	4.4	4.8	< LOD
Carmencita	39.8	40.2	< LOD	1.5	60.2	58.3
Tanzania	26.3	< LOD	< LOD	< LOD	73.7	100.0
Gibsonii	30.8	21.5	2.0	< LOD	67.2	78.5
Sanguineus	6.0	< LOD	33.3	23.0	60.7	77.0
India	13.8	13.0	25.8	24.3	60.4	62.7
Impala	5.9	< LOD	17.6	6.5	76.5	93.5

Table S13. Proportions in % of two amino acid variants (E138 and D138) of the A-chain of ricin. Data of tryptic peptides generated by RPLC-HDMSE are listed in columns labelled with peptides. Values shown in columns labelled with A-chain were calculated using the mass spectrum of intact reduced and alkylated A-chain of ricin. Values below the lower limit of detection were indicated as < LOD.

**Sample**	**A-Chain E138**		**A-Chain D138**	
	Peptides	A-Chain	Peptides	A-Chain
Zanzibariensis 1	61	56	39	44
Zanzibariensis 2	64	57	36	43
Zanzibariensis 2	65	58	35	42
Carmencita D	100	100	< LOD	0
Carmencita E	100	100	< LOD	0
Carmencita	100	100	< LOD	0
Tanzania	62	53	38	47
Gibsonii	100	100	< LOD	0
Sanguineus	100	100	< LOD	0
India	100	100	< LOD	0
Impala	100	100	< LOD	0
